# Evaluation of polyphenols from *Broussonetia papyrifera* as coronavirus protease inhibitors

**DOI:** 10.1080/14756366.2016.1265519

**Published:** 2017-01-22

**Authors:** Ji-Young Park, Heung Joo Yuk, Hyung Won Ryu, Su Hwan Lim, Kyung Su Kim, Ki Hun Park, Young Bae Ryu, Woo Song Lee

**Affiliations:** a Natural Product Material Research Center, Korea Research Institute of Bioscience and Biotechnology, Jeongeup, Republic of Korea;; b Natural Medicine Research Center, Korea Research Institute of Bioscience and Biotechnology, Ochang, Republic of Korea;; c Division of Applied Life Science (BK21 program, IALS), Graduate School of Gyeongsang National University, Jinju, Republic of Korea

**Keywords:** *Broussonetia papyrifera*, coronavirus, papain-like protease, polyphenol, SARS

## Abstract

The current study was designed to assess the inhibitory activity of *Broussonetia papyrifera*-derived polyphenols against 3-chymotrypsin-like and papain-like coronavirus cysteine proteases. The isolated compounds were broussochalcone B (1), broussochalcone A (2), 4-hydroxyisolonchocarpin (3), papyriflavonol A (4), 3′-(3-methylbut-2-enyl)-3′,4,7-trihydroxyflavane (5), kazinol A (6), kazinol B (7), broussoflavan A (8), kazinol F (9), and kazinol J (10). All polyphenols were more potent against papain-like protease (PL^pro^) than against 3-chymotripsin-like protease (3CL^pro^); therefore, we investigated their structural features that were responsible for this selectivity. Compound **4** was the most potent inhibitor of PL^pro^ with an IC_50_ value of 3.7 μM. The active compounds displayed kinetic behaviors, and the binding constants of their interaction with PL^pro^ were determined from surface plasmon resonance analysis. Our results suggest *B. papyrifera* constituents as promising candidates for development into potential anti-coronaviral agents.

## Introduction

Severe acute respiratory syndrome (SARS), a contagious and often fatal respiratory illness, was first reported in the Guangdong province in China in November 2002. The disease quickly spread to other Asian countries, North America, and Europe, infecting more than 8000 individuals and causing approximately 800 deaths^1^. Recently, just as SARS began to fade from public memory, a new coronavirus called Middle East respiratory syndrome coronavirus (MERS-CoV) emerged in 2012. Pathogenic human CoVs, such as MERS-CoV and SARS-CoV, killed approximately 36% of infected patients in Saudi Arabia and South Korea^2–4^. It is alarming the new and deadly CoVs can emerge at any time with the potential to become pandemics. Unfortunately, neither a protective vaccine nor an effective antiviral agent for the prevention and treatment of SARS is available. Therefore, continued development of therapeutic and prophylactic countermeasures against potentially deadly CoVs is warranted. The SARS-CoV is a crown-like enveloped virus that contains a positive-strand RNA whose genome sequence exhibits only moderate homology to other known CoVs^5^. Since SARS-CoV proteases (3-chymotrypsin-like protease [3CL^pro^] and papain-like protease [PL^pro^]) are synthesized as large precursor proteins that are cleaved to generate mature active proteins and their structures are conserved across the CoV genera, targeting these proteins is an effective antiviral strategy in suppressing viral genome replication to cure CoV infection^1^^,^ 6.

As a continuation of our interest in natural product-derived anti-CoV candidates, we report in this study, the inhibitory actions of polyphenols from *Broussonetia papyrifera* against CoV proteases. We have previously reported that the α-glucosidase inhibitory activity of polyphenols from this genus depends on the number of prenyl groups in the molecule^7^. Many natural inhibitors of various glucosidases have been explored for potential application as medicines for carbohydrate-mediated diseases such as cancer^8^, diabetes^9^, and infectious diseases. Endoplasmic reticulum α-glucosidases are responsible for the stepwise removal of terminal glucose residues from *N*-glycan chains attached to nascent glycoproteins. These reactions are the first steps in *N*-linked glycan processing and are essential for the proper folding and function of many glycoproteins^10^. Because most viral envelope glycoproteins contain *N*-linked glycans, α-glucosidase inhibitors such as 1-deoxynojirimycin and castanospermine have been proposed as potentially useful broad-spectrum antiviral agents based on their activity on a variety of enveloped viruses^11^^,^ 12.

We were encouraged by these preliminary findings and focused our attention on investigating the antiviral activities of polyphenols on CoV cysteine proteases. Here, we characterized and evaluated the anti-SARS/MERS activity of the following *B. papyrifera* root-derived glucosidase inhibitor: broussochalcone B^1^, broussochalcone A^2^, 4-hydroxyisolonchocarpin^3^, papyriflavonol A^4^, 3′-(3-methylbut-2-enyl)-3′,4′,7-trihydroxyflavane^5^, kazinol A^6^, kazinol B^7^, broussoflavan A^8^, kazinol F^9^ and kazinol J^,^ 7 ^,^ 10 ^,^ 13. Preliminary biochemical screening of 14 naturally derived compounds (comprising authentic and in-house compounds, as well as isoliquiritigenin, kaempferol, quercetin, and quercetin-β-galactoside^14^) were performed to assess their potential as novel CoV cysteine protease inhibitors *in vitro*. The present results indicate that some of these compounds may serve as lead compounds for the development of CoV protease inhibitors and can be used to elucidate the structure–function relationships of varied polyphenols.

## Materials and methods

### Plant materials

Roots of *B. papyrifera* were collected at Gonyang in Sacheon, South Korea, in July 2008 and were identified by Prof. Myong Gi Chung at the Herbarium of Gyeongsang National University (GNUC). A voucher specimen (KHPark 210709) was deposited at GNUC.

### Chemicals and materials

Nuclear magnetic resonance (NMR) spectra were analyzed using a Bruker AM 500 spectrometer (Bruker, Karlsruhe, Germany), with the chemical shifts represented in parts per million (ppm, *δ*). Thin layer chromatography (TLC) was performed on precoated silica-gel 60 F254 plates and RP-C18 F254s plates (20 × 20 cm; Merck, Darmstadt, Germany), and spots were visualized by UV illumination (254 nm) or by spraying with 10% H_2_SO_4_ in ethanol followed by heating. Solvents for extraction and isolation were purchased from SK Chemicals Co. (Seoul, South Korea) and were redistilled before use. Deionized water was prepared from ultrapure water processed by a Milli-Q purification system (18.2 MΩ; Millipore, Billerica, MA). Solvents for NMR experiments (CDCl_3_, (CD_3_)_2_CO, and CD_3_OD) were purchased from Cambridge Isotope Laboratories Inc. (Andover, MA).

### Extraction and isolation

The dried roots of *B. papyrifera* (10.0 g) were extracted with ethanol (4.0 L, three times) at room temperature and evaporated using a rotary evaporator at temperatures below 45 °C to obtain the total extract (143.0 g). A portion of this residue (5 g) was fractionated on a reversed-phase (RP) silica gel column (5 × 30 cm, YMC ODS AQ-HG 10 μM, 220 g) using a Puriflash 450 medium-pressure liquid chromatography (MPLC) system (Interchim, Montluçon, France) with a linear gradient of 0% to 100% CH_3_OH/H_2_O and 30 mL/min flow rate to generate nine fractions (BP Fr. 1–9). BP Fr. 6 and 7, which exhibited the most potent anticoronaviral activity among the fractions, were selected for isolation of active compounds. BP Fr. 6 (456.5 mg) was isolated by preparative high-performance liquid chromatography (prep-HPLC) using a PLC 2020 personal purification system (Gilson, Inc., Middleton, WI) with a Waters BEH C18 column (Milford, MA) [H_2_O:ACN, 20 →80% (45 min); 80 →98% (10 min); flow rate: 18 mL/min] by repeated injection of 10 mg/mL MeOH dilutions to give compounds **1** (107.0 mg), **2** (181.0 mg), **3** (8.0 mg), **5** (41.0 mg), and **8** (13.0 mg). BP Fr. 7 (575.0 mg) was subjected to prep-HPLC using a Waters BEH C18 column [H_2_O:ACN, 4 0 →98% (45 min); 98% (10 min); flow rate: 18 mL/min] by repeated injections of 10 mg/mL MeOH dilutions to give compounds **4** (6.4 mg), **6** (115.0 mg), **7** (88.0 mg), **9** (49.6 mg) and **10** (35.8 mg). All isolated compounds were identified based on their spectroscopic data.

### UPLC-Q-TOF-MS analysis

The ethanol extracts and subfractions were analyzed by ultra-performance liquid chromatography-photodiode array-quadrupole-time of flight-mass spectrometry (UPLC-PDA-Q-TOF-MS). Chromatographic separations were performed on a 2.1 × 100 mm, 1.7 μm Waters ACQUITY BEH C18 chromatography column at 35 °C. The mobile phases A and B were water with 0.1% formic acid and acetonitrile with 0.1% formic acid, respectively. The solvent gradient was programed as follows: 0 − 1 min, 10% B; 1 − 12 min, 10 − 98% B; and wash to 13.5 min with 98% B. The recycle time was 1.5 min, and the flow rate was 0.4 mL/min.

The mass spectrometer was operated in positive ion mode. N_2_ was used as the desolvation gas. The desolvation temperature was set to 350 °C, the flow rate to 500 L/h, and the source temperature to 100 °C. The capillary and cone voltages were set to 2300 and 35 V, respectively. A Waters Q-TOF Premier^TM^ spectrometer was operated in V mode with a mass resolving power of 9000. Data were collected for each test sample from 100 to 1500 Da with a 0.25-s scan time and a 0.01-s interscan delay over a 15-min analysis time. Leucine–enkephalin was used as the reference compound (*m/z* 554.2615 in the negative mode) at an infusion flow rate of 1 μL/min.

### Expression, purification and preparation of SARS-CoV viral proteases

SARS-CoV 3CL^pro^ and PL^pro^ were prepared according to previously described methods^15–17^. For expression of His-tagged SARS-CoV 3CL^pro^ and PL^pro^, recombinant plasmids were transformed into *Escherichia coli* BL21 (DE3) CodonPlus-RIL competent cells (Stratagene, La Jolla, CA). The purified proteases were stored at −80 °C before use in any of the assays described below^15^^,^ 16.

#### Expression, purification and preparation of MERS-CoV viral proteases

Sequences of MERS-CoV PL^pro^ (GenBank accession number KF192507.1: polyprotein residues 1436–1803) and MERS-CoV 3CL^pro^ (GenBank accession number KF192507.1: polyprotein residues 3247–3553) were synthesized (BIONEER Co., Daejeon, South Korea), digested by NheI-XhoI and then inserted into the pET-28a(+) vector (Novagen), which contained a 6 × His-tag at the C-terminus (Figure S32, S33). The constructed plasmids were transformed into *E. coli* BL21 (DE3) HIT competent cells (Real Biotech Co., Taipei, Taiwan) that were streaked on a LB agar plate containing 50 μg/mL kanamycin at 37 °C. For large-scale protein expression, cultures were grown in 0.5 L of LB medium at 37 °C to an absorbance of 0.5 at 600 nm, induced by overnight incubation with 0.5 mM isopropyl β-d-thiogalactopyranoside (IPTG) at 16 °C. After centrifuging at 6000 *g* at 4 °C for 20 min, the cell pellets were suspended in buffer A (20 mM sodium phosphate, 300 mM NaCl, 20 mM imidazole, and 0.1% Triton X-100, pH 7.5) and then lysed by sonication. The crude extracts were then centrifuged at 12,000 rpm for 30 min to remove the insoluble pellet. The supernatant was loaded onto a Ni-Sepharose column equilibrated with buffer A. The column was washed with buffer A and the protein sample was eluted with 100–300 mM imidazole. The elution buffer was then changed to 20 mM Tris-HCl buffer (pH 7.5) and the protein was concentrated using an Amicon Ultra filter with a 10,000 molecular weight cutoff (Merck Millipore Ltd, Carrigtwohill, Ireland). The enzyme concentration was determined from its absorbance at 280 nm. The purity and molecular weights of MERS-CoV 3CL^pro^ and MERS-CoV PL^pro^ were verified by sodium dodecyl sulfate polyacrylamide gel electrophoresis; the purified proteases were approximately 36 kDa (3CL^pro^) and 33 kDa (PL^pro^). The kinetic parameters of their enzymatic activity were analyzed with fluorescent substrates, whose concentration ranged from 1.25 to 100 μM. The *K*_m_ values were 2.7 ± 0.3 μM (MERS-CoV 3CL^pro^) and 50.9 ± 5.3 μM (MERS-CoV PL^pro^) (Figure 2). The purified proteins were stored at −80 °C before use in any of the assays described below.

### MERS-CoV 3CL^pro^ assay

The enzyme activity of MERS-CoV 3CL^pro^ was measured by cleavage of the synthetic 11-mer peptide Dabcyl-TSGVLQSGLVK-Edans and the nsp4/5 site of the MERS-CoV 3CL^pro^ polyprotein using the fluorescent resonance energy transfer (FRET) method. The peptide was synthesized by Anygen Co. (Gwangju, South Korea) to approximately 95% purity as determined by HPLC on a RP C18 column. The cleavage activity of the enzyme was assayed by incubating 80 nM MERS-CoV 3CL^pro^ and 10 μM of the peptide substrate for 60 min at 37 °C in a total volume of 200 μL in 20 mM sodium phosphate buffer (containing 300 mM NaCl, pH 6.0). The enhanced fluorescence due to the protease-catalyzed substrate cleavage was measured at an excitation wavelength at 360 nm and an emission wavelength of 590 nm using a fluorescence plate reader (Flx800; BioTeck Instrument Inc., Winooski, VT).

For MERS-CoV 3CL^pro^ inhibition assay with the isolated compounds, 80 nM MERS-CoV 3CL^pro^ and 0–200 μM of the individual compounds were mixed with the substrate (10 μM) at 37 °C, and the fluorescence intensity was monitored. The enzyme activity (from fitting of experimental data to the logistic curve of Equation (1) was determined by using a time-drive protocol with the initial velocity recorded over a range of concentrations. The data were analyzed by nonlinear regression using Sigma Plot 10.0 (Systat Software Inc., San Jose, CA).

(1) $$\mathrm{Activity}\;\left(\%\right)=[1/(1+([I]/{\mathrm{IC}}_{50}))]\times 100$$

### MERS-CoV PL^pro^ assay

The enzyme activity of MERS-CoV PL^pro^ was measured by modifying the SARS-CoV PL^pro^ assay that we previously described^15^. The fluorogenic peptide Arg-Leu-Arg-Gly-Gly-AMC (ENZO Life Sciences, Farmingdale, NY) and 300 nM purified MERS-CoV PL^pro^ in 20 mM sodium acetate buffer (pH 5.5) were used as the substrate and enzyme, respectively. For inhibition studies, 300 nM MERS-CoV PL^pro^ and 0–200 μM of the individual compounds were mixed with the substrate (50 μM) at 37 °C. The fluorescence intensity was monitored at excitation and emission wavelengths of 360 and 460 nm, respectively, on a SpectraMax M^2e^ multimode microplate reader (Molecular Devices, Sunnyvale, CA).

### SARS-CoV viral proteases inhibition assay

The inhibitory effect of each compound on SARS-CoV 3CL^pro^ activity was measured using the FRET method^18^. In this assay, the 14-mer fluorogenic peptide Dabcyl-KTSAVLQSGFRKME-Edans (Anygen Co., Republic of Korea) was used as a substrate and enzyme activity was determined by measuring the increase in fluorescence intensity by continuously monitoring the reaction at 590 nm after excitation at 360 nm using a fluorescence plate reader (Flx800; BioTeck Instrument Inc.) for up to 60 min. The reaction mixture contained 10 μg/mL SARS-CoV 3CL^pro^ (final concentration, 2.5 μg), the test compounds (0 to 200 μM), and 10 μM of the fluorogenic 14-mer peptide substrate in 20 mM Bis-Tris buffer (pH 7.5).

The activity of SARS-CoV PL^pro^ was measured using a method previously described by our group^15^^,^ 16. The inhibition assay was optimized in a 96-well plate to establish suitable assay conditions and incubation times. The fluorogenic peptide Arg-Leu-Arg-Gly-Gly-AMC (ENZO Life Sciences, Farmingdale, NY), and 208 nM purified SARS-CoV PL^pro^ in 20 mM Tris-HCl buffer (pH 6.8) were used as the substrate and the enzyme, respectively. For inhibition studies, 54 nM SARS-CoV PL^pro^ and 0–200 μM of the individual compounds were mixed with the substrate (50 μM) at 37 °C. The fluorescence intensity was monitored at excitation and emission wavelengths of 360 and 460 nm, respectively, on a SpectraMax M^2e^ multimode microplate reader (Molecular Devices). 

To study the kinetics of SARS-CoV PL^pro^ inhibition by compounds **1–10**, various concentrations of these compounds were mixed with SARS-CoV PL^pro^ in assay buffer containing a predetermined substrate. The inhibition constant, *K*_i_ was calculated by nonlinear regression analysis by fitting different models of enzyme inhibition to the kinetic data using SigmaPlot Enzyme Kinetics Module 1.3 (Systat Software Inc., San Jose, CA). The inhibition mechanism of the compounds was determined by comparing the statistical results, which included Akaike information criterion corrections (AICc), with different inhibition models and selecting the one with the best fit^19^.

### SARS-CoV PL^pro^ deubiquitination assay

The fluorogenic substrate Ub-AMC (Ubquitin-7-amino-4-methylcoumarin) (Enzo Life Sciences) added at a concentration of 100 nM to 20 mM Tris-HCl buffer (pH 6.8) was used for deubiquitination assays as previously described^15^. The enzymatic activity at 37 °C was determined by continuously monitoring the fluorescence emission and excitation wavelengths at 360 and 460 nm, respectively, with a SpectraMax M^2e^ multimode microplate reader (Molecular Devices Co., Sunnyvale, CA). The release of AMC was measured in the same manner as described previously^20^^,^ 21.

### SARS-CoV PL^pro^ deISGylation assay

The SARS-CoV PL^pro^ inhibition assay with interferon-stimulated gene 15 (ISG15)-AMC (Boston Biochem, Cambridge, MA) was performed in a 96-well plate at 37 °C in 20 mM Tris-HCl buffer (pH 6.8) containing 54 nM SARS-CoV PL^pro^ and various concentrations of the compounds ranging from 1 to 200 μM. The ISG15-AMC substrate concentration was 50 nM. The release of AMC was monitored at excitation and emission wavelengths of 360 and 460 nm, respectively^20^.

### Real-time analysis of ligand interactions with proteases by surface plasmon resonance (SPR)

Surface plasmon resonance (SPR) measurements were performed on a Reichert SPR SR7500DC instrument (Depew, NY), which was kindly provided on loan from WOOJUNG BSC, Inc. (Suwon-si, Korea). The enzyme was dialyzed in phosphate-buffered saline (PBS, pH 7.4) prior to immobilization. The surface of dextran-coated chips (CMDH, carboxymethyldextran hydrogel chip) was activated with a 7-min injection of EDC/sulfo-NHS mixture at 200/50 mM concentration. The protein was diluted to 250 μg/mL using 10 mM sodium acetate buffer (pH 4.0) and was injected for 40 min. The active sites were quenched with 1 M ethanolamine buffer (pH 8.0) and washed with PBS (pH 7.4). The binding surface was stabilized with 3–5 blank injections of PBS, which was used as the running and sample dilution buffers in all experiments. The tested inhibitors were first prepared as 20 mM stock solutions in dimethyl sulfoxide (DMSO) and further diluted with PBS immediately prior to the experiments. The binding reaction was analyzed by automated runs of a twofold dilution series and two zero concentration injections. Regeneration steps were performed using 10 mM NaOH. The data was analyzed with the Scrubber2 software. Reference and blank binding dissociation curves were fit globally to a two-state binding kinetics model^22^.

## Results and discussion

**Figure 1.**
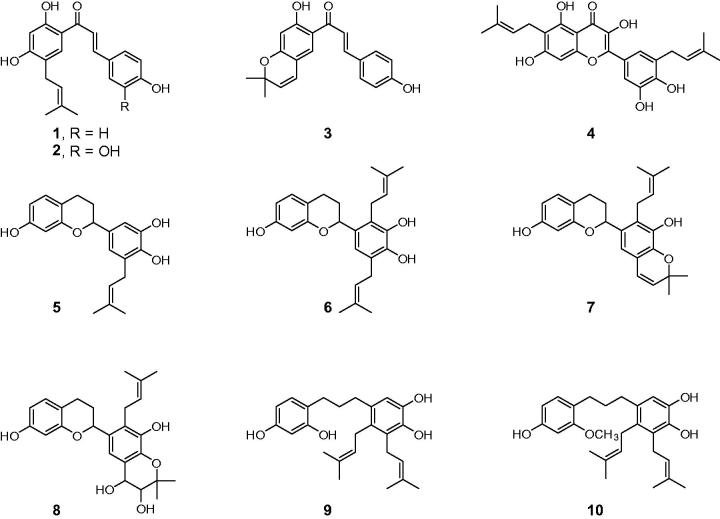
Chemical structures of isolated compounds from *Broussonetia papyrifera*.

The high potency of the chloroform extract (IC_50 _=_ _9.3 μg/mL against α-glucosidase) indicated the presence of compounds that may by therapeutic against infectious diseases^7^. Therefore, the major compounds of BP Fr. 6 and 7 were expected to exhibit enzyme inhibitory activities. UPLC-PDA-QTOF-MS analyses were carried out using a C18 column with a linear gradient of acetonitrile/water. Each peak was characterized using MS. The *m*/*z* values of molecular ions [M-H]^−^ of compounds **1–10** were 323, 339, 321, 437, 325, 393, 391, 425, 395, and 409, respectively (Supplementary material). We isolated ten active compounds using prep-HPLC on octadecyl-functionalized silica gel. The isolated compounds were characterized by comparison of spectroscopic data, including 1D and 2D-NMR, mass spectral data of molecular ions, UV–Vis absorption maxima, and optical rotation, with those published previously^7^^,^ 13. Compounds **1**–**10** were identified as broussochalcone B^1^, broussochalcone A^2^, 4-hydroxyisolonchocarpin^3^, papyriflavonol A^4^, 3′-(3-methylbut-2-enyl)-3′,4′,7-trihydroxyflavane^5^, kazinol A^6^, kazinol B^7^, broussoflavan A^8^, kazinol F^9^ and kazinol J^10^ (Figure 1).

**Figure 2.**
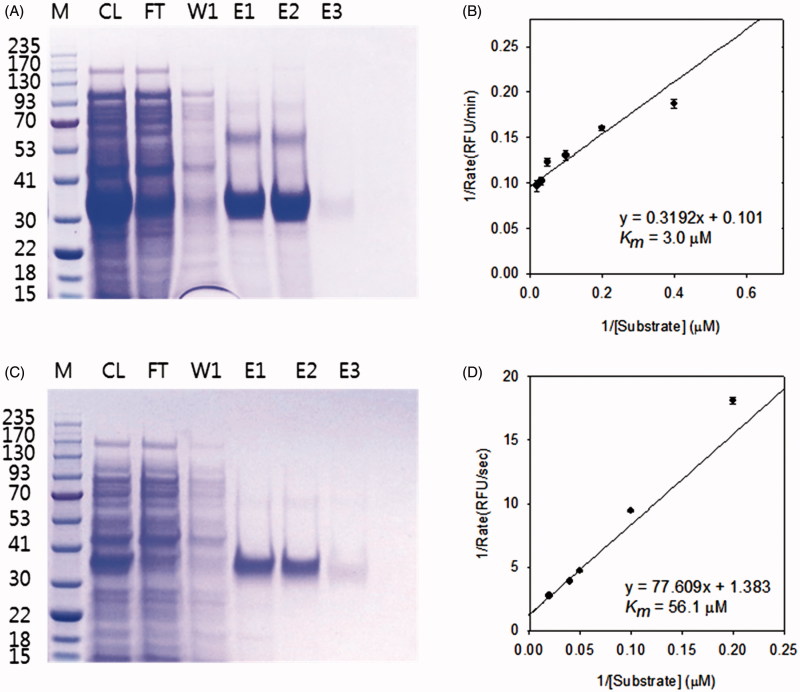
SDS-PAGE of purified MERS-CoV 3CL^pro^ (A) and MERS-CoV PL^pro^ (C); Lineweaver–Burk plots for the determination of *K_m_* values against the MERS-CoV 3CL^pro^ (B) and MERS-CoV PL^pro^ (D). (A, C) M, protein molecular-weight markers (kDa); CL, cell lysate; FT, flow-through; W1, 20 mM imidazole wash; E1, E2 and E3, 50, 100 and 200 mM imidazole elution. (B, D) The reaction was done at various substrate concentrations to obtain *K*_m_ value of the enzyme. SigmaPlot was used to fit the kinetic data using Lineweaver–Burk double reciprocal plots.

**Figure 3.**
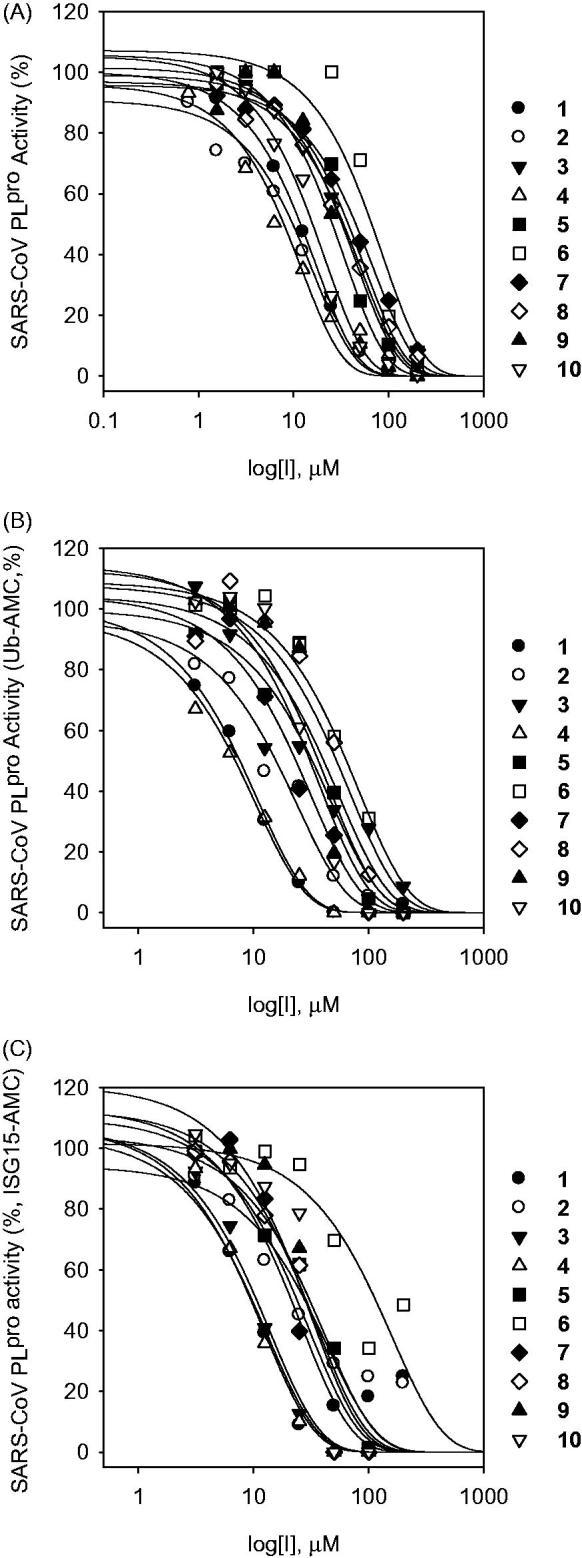
Effects of compounds **1–10** on the activity of SARS-CoV PL^pro^ for proteolysis of substrates (A; LXGG-AMC, B; Ubiqutin-AMC, C; ISG15-AMC).

We first expressed CoV cysteine proteases (SARS-3CL^pro^, SARS-PL^pro^, MERS-3CL^pro^, and MERS-PL^pro^) in *E. coli*, purified the proteins to homogeneity, and determined their activity with a fluorometric cleavage assay. The IC_50_ values of the isolated compounds, which measure their ability to inhibit the catalytic activity of the proteases, were calculated by fitting the data to a logic derivative equation. We also examined the α-glucosidase inhibitory capacity of the isolated compounds^7^. These compounds showed dose-dependent inhibitory effects on both α-glucosidase and cysteine proteases. Results of the CoV cysteine protease inhibition assays are summarized in Tables 1 and 2. All tested polyphenols (**1–10**) showed dose-dependent inhibitory effect on SARS-CoV PL^pro^ (Figure 3). As shown in Table 1, the compounds were more potent against SARS-CoV PL^pro^ compared with the other cysteine proteases. The IC_50_ values of compounds **1–10** against *trans*-cleavage activity of SARS-CoV 3CL^pro^ ranged from 30.2 to 233.3 μM. Among the isolated polyphenols, the C5-alkyl group (prenyl)-substituted flavan^5^ exhibited the most potent inhibitory effect against SARS-CoV 3CL^pro^ and was more effective than quercetin (IC_50 _=_ _52.7 μM). General structural–activity relationships can be deduced from these data, for example, molecules with a C4-OH group and a saturated chromenone with a dihydroxy group at ring C showed higher inhibitory activity than those with a closed prenyl group (**7**, IC_50 _=_ _233.3 μM). Chalcones (**1–3**, IC_50_: 57.8–202.7 μM) and biphenyl propanoids (**9** and **10**, IC_50_: 43.3–64.2 μM) moderately inhibited SARS-CoV 3CL^pro^ activity. Furthermore, the activity of the compounds was significantly affected by subtle changes in their molecular structure, such as from prenylation and methylation (*vide infra*).

**Table 1.** Inhibitory effects of isolated polyphenols^1–10^ and commercial polyphenols on SARS-CoV cysteine proteases.

		SARS-CoV PL^pro^, IC_50_ (μM)			
		LRLRGG-AMC			
Compounds	SARS-CoV 3CL^pro^,IC_50_ (μM)^a^	IC_50_ (μM)	Inhibition type (*K*_i_, μM)	Deubiquitinationactivity	DeISGylationactivity
**1**	57.8 ± 0.5	11.6 ± 0.7	Noncompetitive (6.6 ± 0.5)	8.9 ± 0.8	10.2 ± 1.4
**2**	88.1 ± 13.0	9.2 ± 1.5	Noncompetitive (8.0 ± 0.4)	21.8 ± 1.8	12.6 ± 1.8
**3**	202.7 ± 3.9	35.4 ± 11.3	Noncompetitive (27.7 ± 1.7)	30.9 ± 5.7	28.8 ± 3.3
**4**	103.6 ± 17.4	3.7 ± 1.6	Noncompetitive (5.9 ± 0.4)	7.6 ± 0.4	8.5 ± 1.2
**5**	30.2 ± 6.8	35.8 ± 6.7	Noncompetitive (15.9 ± 0.8)	41.4 ± 3.0	34.2 ± 1.4
**6**	84.8 ± 10.4	66.2 ± 6.8	Noncompetitive (40.5 ± 3.4)	74.8 ± 5.7	70.8 ± 10.5
**7**	233.3 ± 6.7	31.4 ± 2.9	Noncompetitive (36.7 ± 2.7)	21.4 ± 0.2	21.5 ± 3.8
**8**	92.4 ± 2.1	30.4 ± 5.5	Noncompetitive (23.4 ± 1.6)	59.9 ± 0.5	52.1 ± 6.3
**9**	43.3 ± 10.4	27.8 ± 2.5	Noncompetitive (12.1 ± 0.7)	45.2 ± 5.5	31.2 ± 3.2
**10**	64.2 ± 1.7	15.2 ± 1.6	Noncompetitive (10.7 ± 0.9)	33.3 ± 0.2	30.3 ± 2.4
Isoliquiritigenin	61.9 ± 11.0	24.6 ± 1.0	Noncompetitive (23.0 ± 0.9)	17.2 ± 2.3	12.6 ± 0.7
Kaempferol	116.3 ± 7.1	16.3 ± 2.1	Noncompetitive (13.7 ± 0.8)	61.7 ± 3.8	71.7 ± 7.4
Quercetin	52.7 ± 4.1	8.6 ± 3.2	Noncompetitive (7.0 ± 0.7)	20.7 ± 2.0	34.4 ± 2.6
Quercetin-β-galactoside	128.8 ± 4.5	51.9 ± 5.5	Noncompetitive (56.1 ± 2.5)	136.9 ± 4.7	67.7 ± 8.4

a All compounds were examined in a set of experiments repeated three times; IC_50_ values of compounds represent the concentration that caused 50% enzyme activity loss.

**Table 2.** MERS CoV proteases (3CL^pro^ and PL^pro^) inhibitory activity of polyphenols^1–10^ and commercial polyphenols

Compounds	MERS-CoV 3CL^pro^,IC_50_ (μM)^a^	MERS-CoV PL^pro^,IC_50_ (μM)
**1**	27.9 ± 1.2	112.9 ± 10.1
**2**	36.2 ± 0.4	42.1 ± 5.0
**3**	193.7 ± 15.6	171.6 ± 10.2
**4**	64.5 ± 4.9	112.5 ± 7.3
**5**	34.7 ± 2.0	48.8 ± 6.6
**6**	NA^b^	88.5 ± 3.9
**7**	NA	94.9 ± 13.1
**8**	125.7 ± 17.4	49.1 ± 7.5
**9**	135.0 ± 5.1	39.5 ± 5.1
**10**	109.2 ± 3.7	55.0 ± 1.3
Isoliquiritigenin	33.9 ± 7.7	82.2 ± 7.7
Kaempferol	35.3 ± 5.3	206.6 ± 1.7
Quercetin	34.8 ± 1.2	NA
Quercetin-β-galactoside	68.0 ± 2.4	129.4 ± 14.5

a All compounds were examined in a set of experiments repeated three times; IC_50_ values of compounds represent the concentration that caused 50% enzyme activity loss.

b No activity.

The prenylated flavone derivative, **4**, exhibited the highest inhibitory effects on SARS-CoV PL^pro^ (IC_50 _=_ _3.7 μM). The positions of the two prenyl groups had substantial effect on SARS-CoV PL^pro^ inhibition by the corresponding compounds. The SARS-CoV PL^pro^ inhibitory potency of the prenyl flavone was considerably higher than that of flavone derivatives, including keampferol (IC_50 _=_ _16.3 μM), quercetin (IC_50 _=_ _8.6 μM), and quercetin-β-galactoside (IC_50 _=_ _51.9 μM). It is probable that the presence of a prenyl group within the resorcinol group allows for the formation of strong hydrophobic interactions with the enzyme. Additionally, an increase in the number of hydroxyl groups in the flavone backbone resulted in higher inhibitory activity, which can be noted from the lower IC_50_ value of quercetin (8.6 μM) compared to that of kaempferol (IC_50 _=_ _16.3 μM). Among chalcone derivatives with a closed prenyl ring (chromenone, which is characterized by a C5-prenyl group in ring B), diminishing protease inhibitory activity was observed (**2**, IC_50 _=_ _9.2 μM >**1**, IC_50 _=_ _11.6 μM > isoliquiritigenin, IC_50 _=_ _24.6 μM>**3**, IC_50 _=_ _35.4 μM). In contrast, the number of prenyl groups and the degree of saturation did not have any effect on the prenylated flavans. As shown in Table 1, all flavans except **6** exhibited moderate inhibitory activity (IC_50_ values ranging from 30.4 to 35.8 μM). Additionally, compound **10**, which contains a methoxy group that is also present in the corresponding biphenyl propanoids, was two-fold more effective (IC_50_ = 15.2 μM) than the unmethylated **9**.

**Figure 4.**
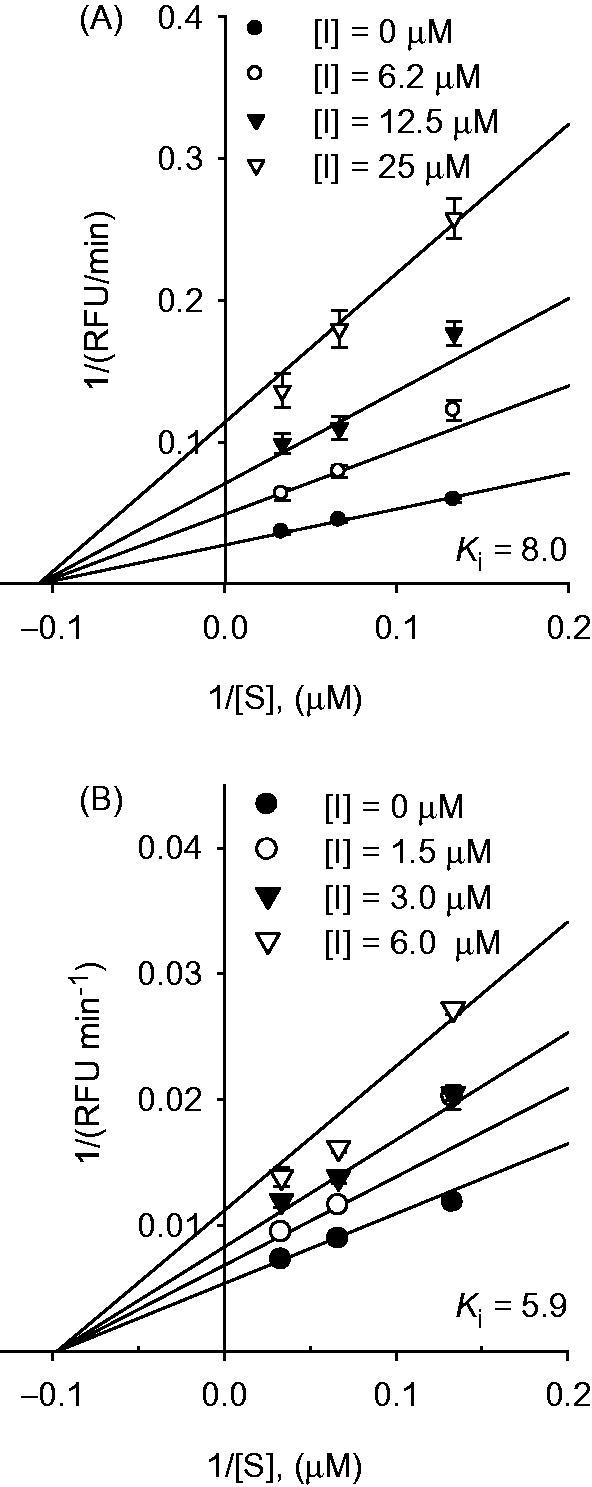
Graphical determination of the inhibition type for compounds. (A) Lineweaver–Burk plot for the inhibition of compound **2** on SARS-CoV PL^pro^. (B) Lineweaver–Burk plot for the inhibition of compound **4** on SARS-CoV PL^pro^.

To investigate the protease inhibition mechanism, we examined the effect of the compounds (**1–10** and corresponding compounds) on the kinetics of substrate proteolysis. The kinetic studies showed that compounds **2** and **4** exhibited noncompetitive inhibition based on Lineweaver–Burk plots (1/*V* vs. 1/[*S*]) with the same *x*-axis intercept (Figure 4). Inhibition of SARS-CoV PL^pro^ activity decreased with increasing concentrations of the substrate. All inhibitors studied exhibited the same mode of inhibition. The *K*_i_ values of the compounds were calculated from Dixon plots (Table 1).

**Figure 5.**
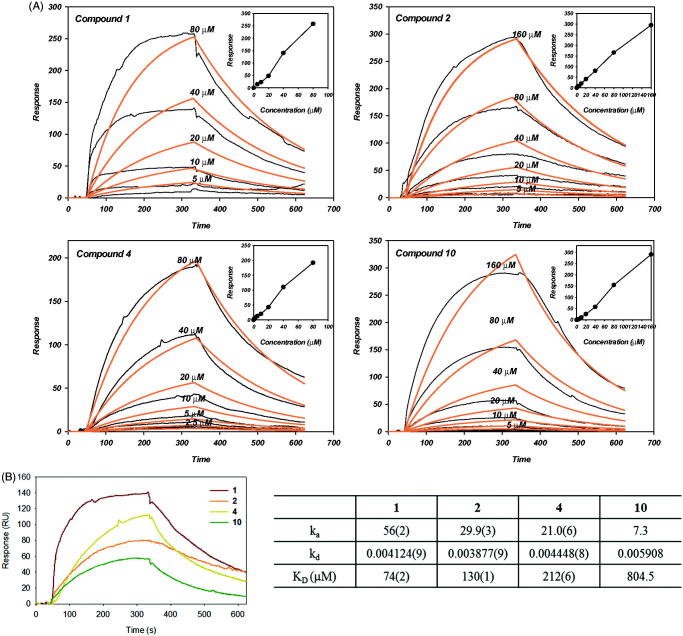
Surface plasmon resonance analysis for the interaction of compounds with SARS-CoV PL^pro^ (A) Normalized refractive index change obtained for different compounds (**1**, **2**, **4** and **10**) concentrations. The experiments were carried out in PBS at a 30 μL/mL flow rate. (insert) The linear dependence of the response in function of the concentration of compounds injected over immobilized SARS-CoV PL^pro^ support the idea of nonspecific direct binding. (B) SPR sensogram of the interaction between compounds of 40 μM and SARS-CoV PL^pro^ (left). The *K*_D_ values for the binding compounds to immobilized SARS-CoV PL^pro^. *k*_a_ and *k*_d_ from where *K*_D_ were calculated are also shown (right).

Surface plasmon resonance is a useful technique for monitoring molecular reactions in real-time and has been used to determine binding specificity as well as rates of association and dissociation between ligands and target proteins. In this study, SARS-CoV PL^pro^ was immobilized on a sensor chip and the most potent PL^pro^ inhibitor, compound **4**, was passed over the sensor’s surface. As shown in Figure 5, the interaction of compound **4** with SARS-CoV PL^pro^ was due to a specific binding event. The SPR sensorgram was recorded at different concentrations of the compound and was used to determine the kinetic-binding parameters, the association rate constant (*k*_a_ μM^−^ ^1^ s^−^ ^1^), and the dissociation rate constant (*k*_d_, s^−^ ^1^). For SARS-CoV PL^pro^, compound **4** increased the SPR sensorgram in a significant and dose-dependent manner. The *K*_D_ (*k*_d_/*k*_a_) for the interaction between compound **4** and SARS-CoV PL^pro^ indicated a classical interaction, with *k*_a_ = 21.0 μM^−^ ^1^ s^−^ ^1^, *k*_d_ = 0.004448 s^−1^, and *K*_D_ = 212 μM. Although inhibition assays revealed compound **2** as the second most potent inhibitor of SARS-CoV PL^pro^, its *K*_D_ was higher than that of compound **4**.

In recent year, PL^pro^ has been the focus of numerous studies on the development of chemotherapeutic drugs against CoV-induced diseases. It is part of the nsp3 protein, and cleaves at the nsp1/2, nsp2/3 and nsp3/4 boundaries by recognizing the LXGG consensus motif. In addition, PL^pro^ exhibits deubiquitinating (DUB) activity and antagonizes the induction of type-1 interferon (IFN). Interferon-stimulated gene 15 is one of the most upregulated genes upon type I IFN stimulation and is involved in tagging newly synthesized proteins during an antiviral response. Both ubiquitin and ISG15 may have important implications in viral replication and pathogenesis. SARS-CoV PL^pro^ can cleave ubiquitin and ISG15 from cellular conjugates. Similarly, MERS-CoV PL^pro^ has also been shown to possess DUB and deISG15ylating activities. Thus, an effective anti-CoV drug is one that inhibits viral protease activity as well as DUB and deISG15ylating activities. Since **4** inhibits SARS-CoV PL^pro^, we postulated that its efficacy would be higher by having DUB and deISG15ylating activities. Compound **4** strongly inhibited the cleavage of both ubiquitin and ISG15 (IC_50_ values of 7.6 and 8.5 μM, respectively). Complete substitution of prenyl groups (**4** vs. quercetin, IC_50_ values of 20.7 and 34.4 μM, respectively) and introduction of hydroxyl groups strongly affected activity (quercetin vs. kaempferol, IC_50_ values of 61.7 and 71.7 μM, respectively). All of the tested compounds display moderate inhibition of *in vitro* cleavage of ubiquitin (IC_50 _=_ _7.6–136.9 μM) and ISG15 (IC_50 _=_ _8.5–71.7 μM) (Table 1). To date, the function of the DUB activity of PL^pro^ *in vivo* is unknown, as is the identity of its *in vivo* cellular substrate. Thus, further investigation is necessary to determine the specificity of its inhibition by measuring the cleavage of ubiquitin-like protein ISG15, which could reveal whether deISGylation by PL^pro^ is a mechanism by which SARS-CoV inactivates IFN-α/β-induced innate immune response.

To determine whether the isolated compounds described earlier are effective inhibitors of MERS-CoV cysteine proteases, their inhibitory potential were assessed using recombinant 3CL and PL proteases expressed in our laboratory. As shown in Table 2, none of the compounds were potent or selective against MERS-CoV cysteine proteases. Compound **1** effectively inhibited MERS-CoV 3CL^pro^ activity (IC_50 _=_ _27.9 μM), similar to its effect against SARS-CoV 3CL^pro^. It also has a comparable mode of action for subtle structural changed. On the other hand, the isolated polyphenols did not show any significant inhibitory activity against MERS-CoV PL^pro^ (IC_50_ values ranging from 39.5 to 171.6 μM). The most potent MERS-CoV PL^pro^ inhibitor was the biphenyl propanoid, compound **9** (IC_50 _=_ _39.5 μM).

## Conclusions

SARS-CoV, and the recently emerged MERS-CoV, are able to cross species barriers and spread rapidly among humans. In this study, we assessed the inhibitory activity of polyphenols derived from *B. papyrifera* against CoV cysteine proteases. All isolated polyphenols (**1–10**) markedly inhibited both 3CL and PL CoV proteases. The IC_50_ values of these compounds, though higher than those of peptide-derived inhibitors, were still in the low micromolar range. In particular, the isolated compounds exerted significant SARS-CoV PL^pro^ inhibitory activity through noncompetitive inhibition. The prenylated quercetin derivative **4**, showed the most potent PL^pro^ inhibitory activity (IC_50 _=_ _3.7 μM). Additionally, we performed detailed protein-inhibitor SPR-binding analyses of compound **4** with SARS-CoV PL^pro^. Cellular deubiquitination and deISGylation activity of the compounds were also discussed. Based on our enzymatic data showing significant activity of these compounds against CoV proteases, we aim to design more effective CoV inhibitors in future studies.

## Supplementary Material

IENZ_1265519_Supplementary_Material.pdf

## References

[1] Stadler K, Masignani V, Eickmann M, et al SARS-beginning to understand a new virus. Nat Rev Microbiol 2003;1:209–18. 15035025 10.1038/nrmicro775 PMC7097337

[2] Kumar V, Tan KP, Wang YM, et al Identification, synthesis and evaluation of SARS-CoV and MERS-CoV 3C-like protease inhibitors. Bioorg Med Chem 2016;24:3035–42. 27240464 10.1016/j.bmc.2016.05.013 PMC7079562

[3] Chan JF, Lau SK, To KK, et al Middle East respiratory syndrome coronavirus: another zoonotic betacoronavirus causing SARS-like disease. Clin Microbiol Rev 2015;28:465–522. 25810418 10.1128/CMR.00102-14 PMC4402954

[4] Durai P, Batool M, Shah M, Choi S. Middle East respiratory syndrome coronavirus: transmission, virology and therapeutic targeting to aid in outbreak control. Exp Mol Med 2015;47:e181. 26315600 10.1038/emm.2015.76 PMC4558490

[5] Holmes KV. SARS coronavirus: a new challenge for prevention and therapy. J Clin Invest 2003;111:1605–9. 12782660 10.1172/JCI18819 PMC156116

[6] Anand K, Ziebuhr J, Wadhwani P, et al Coronavirus main proteinase (3CLpro) structure: basis for design of anti-SARS drugs. Science 2003;300:1763–7. 12746549 10.1126/science.1085658

[7] Ryu HW, Lee BW, Curtis-Long MJ, et al Polyphenols from *Broussonetia papyrifera* displaying potent alpha-glucosidase inhibition. J Agric Food Chem 2010;58:202–8. 19954213 10.1021/jf903068k

[8] Fernandes B, Sagman U, Auger M, et al β1-6 branched oligosaccharides as a marker of tumor progression in human breast and colon neoplasia. Cancer Res 1991;51:718–23. 1985789

[9] van de Laar FA, Lucassen PL, Akkermans RP, et al α-Glucosidase inhibitors for patients with type 2 diabetes: results from a Cochrane systematic review and meta-analysis. Diabetes Care 2005;28:154–63. 15616251 10.2337/diacare.28.1.154

[10] Chang J, Warren TK, Zhao X, et al Small molecule inhibitors of ER α-glucosidases are active against multiple hemorrhagic fever viruses. Antiviral Res 2013;98:432–40. 23578725 10.1016/j.antiviral.2013.03.023 PMC3663898

[11] Papandreou MJ, Barbouche R, Guieu R, et al The alpha-glucosidase inhibitor 1-deoxynojirimycin blocks human immunodeficiency virus envelope glycoprotein-mediated membrane fusion at the CXCR4 binding step. Mol Pharmacol 2002;61:186–93. 11752220 10.1124/mol.61.1.186

[12] Whitby K, Pierson TC, Geiss B, et al Castanospermine, a potent inhibitor of dengue virus infection *in vitro* and *in vivo* . J Virol 2005;79:8698–706. 15994763 10.1128/JVI.79.14.8698-8706.2005 PMC1168722

[13] Ryu HW, Lee JH, Kang JE, et al Inhibition of xanthine oxidase by phenolic phytochemicals from *Broussonetia papyrifera* . J Korean Soc Appl Biol Chem 2012;55:587–94.

[14] Chen L, Li J, Luo C, et al Binding interaction of quercetin-3-β-galactoside and its synthetic derivatives with SARS-CoV 3CL^pro^: structure-activity relationship studies reveal salient pharmacophore features. Bioorg Med Chem 2006;14:8295–306. 17046271 10.1016/j.bmc.2006.09.014 PMC7125754

[15] Park JY, Jeong HJ, Kim JH, et al Diarylheptanoids from *Alnus japonica* inhibit papain-like protease of severe acute respiratory syndrome coronavirus. Biol Pharm Bull 2012;35:2036–42. 22971649 10.1248/bpb.b12-00623

[16] Park JY, Kim JH, Kim YM, et al Tanshinones as selective and slow-binding inhibitors for SARS-CoV cysteine proteases. Bioorg Med Chem 2012;20:5928–35. 22884354 10.1016/j.bmc.2012.07.038 PMC7127169

[17] Park JY, Ko JA, Kim DW, et al Chalcones isolated from *Angelica keiskei* inhibit cysteine proteases of SARS-CoV. J Enzyme Inhib Med Chem 2016;31:23–30. 10.3109/14756366.2014.1003215 25683083

[18] Park JY, Kim JH, Kwon JM, et al Dieckol, a SARS-CoV 3CL(pro) inhibitor, isolated from the edible brown algae Ecklonia cava. Bioorg Med Chem 2013;21:3730–7. 23647823 10.1016/j.bmc.2013.04.026 PMC7126891

[19] Burnham KP, Anderson DR. Multimodel inference: understanding AIC and BIC in model selection. Sociol Methods Res 2004;33:261–304.

[20] Chou CY, Chien CH, Han YS, et al Thiopurine analogues inhibit papain-like protease of severe acute respiratory syndrome coronavirus. Biochem Pharmacol 2008;75:1601–9. 18313035 10.1016/j.bcp.2008.01.005 PMC7092826

[21] Ratia K, Pegan S, Takayama J, et al A noncovalent class of papain-like protease/deubiquitinase inhibitors blocks SARS virus replication. Proc Natl Acad Sci USA 2008;105:16119–24. 18852458 10.1073/pnas.0805240105 PMC2571001

[22] Nguyen B, Tanious FA, Wilson WD. Biosensor-surface plasmon resonance: quantitative analysis of small molecule-nucleic acid interactions. Methods 2007;42:150–61. 17472897 10.1016/j.ymeth.2006.09.009

