# Supplementary material for: Evaluation of polyphenols from Broussonetia papyrifera as coronavirus protease inhibitors
Source: J Enzyme Inhib Med Chem. 2017 Jan 22;32(1):504–12. doi: 10.1080/14756366.2016.1265519 (PMC6010046; doi:10.1080/14756366.2016.1265519)
Supplement: IENZ_1265519_Supplementary_Material.pdf [file IENZ_A_1265519_SM3052.pdf]

Supplementary Information

Evaluation of polyphenols from *Broussonetia papyrifera* as coronavirus protease inhibitors

Ji-Young Park[1], Heung Joo Yuk[2], Hyung Won Ryu[2], Su Hwan Lim[1], Kyung Su Kim[1], Ki Hun Park[3], Young Bae Ryu[1,*], Woo Song Lee [1,*]

1. Characterization Data

► Spectroscopic data of compounds **1–10**:

- Isolation and determination of the isolated compounds ---------------------- Fig. 1.
- ^{1}H and ^{13}C NMR spectrum, UHPLC-QTOF-MS, HREIMS data, ------------ Fig S1 - S31.

2. Sequence data of MERS-CoV proteases

► Sequence data:

- Sequence data of MERS-CoV 3CL^{pro} -- Fig. S32.
- Sequence data of MERS-CoV PL^{pro} -- Fig. S33.

3. Bioassay Plots

► Graphical data of compounds **1–10**:

- Graphical data of compounds **1-10** against SARS-CoV PL^{pro} --------------- Fig. S34.

*Broussochalcone B (**1**)*: yellow needles; mp 168–169 °C; HRESIMS *m/z* 323.1306 $[M-H]^-$ (calcd for $C_{20}H_{19}O_4$, 323.1283); ^{1}H NMR (500 MHz, acetone–d_6) δ 1.72 (6H, m, H-4″, 5″), 3.27 (2H, d, J = 7.0 Hz, H-1″), 5.34 (1H, s, H-2″), 6.31 (1H, s, H-3′), 6.85 (2H, d, J = 8.1 Hz, H-3, 5), 7.54 (1H, d, J = 15.5 Hz, H-β), 7.59 (2H, d, J = 8.3 Hz, H-2, 6), 7.73 (1H, s, H-6′), 7.76 (1H, d, J = 15.5 Hz. H-α); ^{13}C NMR (125 MHz) δ 16.5 (C-5″), 27.5 (C-4″), 29.4 (C-1″), 101.9 (C-3′), 112.0 (C-1′), 115.5 (C-3, 5), 117.0 (C-α), 120.6 (C-5′), 122.6 (C-2″), 126.4 (C-1), 130.7 (C-2, 6), 130.7 (C-3″), 131.8 (C-6′), 143.9 (C-β), 160.1 (C-4), 163.0 (C-2′), 164.4 (C-4′), 192.0 (C=O).

*Broussochalcone A (**2**)*: yellow needles; mp 182–184 °C; HRESIMS *m/z* 339.1255 $[M-H]^-$ (calcd for $C_{20}H_{19}O_5$, 339.1232); ^{1}H NMR (500 MHz, acetone–d_6) δ 1.58 (3H, s, H-4″), 1.61 (3H, s, H-5″), 3.31 (2H, d, J = 11.5 Hz, H-1″), 5.35 (1H, br t, J = 14.3 Hz. J = 7.2 Hz, H-2″), 6.41 (1H, s, H-3′), 6.92 (1H, d, J = 13.5 Hz, H-5), 7.20 (1H, d, J = 12.0 Hz, H-6), 7.32 (1H, s, H-2), 7.72 (1H, d, J = 15.3 Hz, H-α), 7.72 (1H, δ, J = 15.3 Hz, , H-β), 7.96 (1H, s, H-6′), 13.5 (1H, s, 2-OH); ^{13}C NMR (125 MHz) δ 18.4 (C-5″), 26.3 (C-4″), 29.2 (C-1″), 103.9 (C-3′), 114.8 (C-1′), 116.3 (C-2), 116.9 (C-5), 119.0 (C-α), 121.7 (C-5′), 124.4 (C-2″), 123.8 (C-6), 128.7 (C-1), 132.7 (C-6′), 132.8 (C-3″), 145.7 (C-3), 146.8 (C-β), 149.6 (C-4), 163.9 (C-4′), 166.3 (C-2′), 193.1 (C=O).

*4-Hydroxyisolonchocarpin (**3**)*: amorphous yellow powder; mp 196–197 °C; HRESIMS *m/z* 321.1139 $[M-H]^-$ (calcd for $C_{20}H_{19}O_4$, 321.1127); ^{1}H NMR (500 MHz, CD_3OD) δ 1.38 (6H, s, H-4″, 5″), 5.51 (1H, d, J = 10.0 Hz, H-2″), 6.24 (1H, d, J = 9.6 Hz, H-1″), 6.30 (1H, s, H-3′), 6.82 (2H, s, H-3, 5), 7.34 (1H, d, J = 14.7 Hz, H-α), 7.42 (1H, s, H-6′), 7.48 (1H, s, H-2), 7.48 (1H, s, H-6), 7.74 (1H, d, J = 14.8 Hz, H-β); ^{13}C NMR (125 MHz) δ 29.0 (C-4″, 5″), 78.4 (C-3″), 105.2 (C-3′), 113.9 (C-5′), 114.7 (C-1′), 116.5 (C-3, 5), 118.1 (C-α), 121.5 (C-1″), 126.1 (C-1), 127.8 (C-6′), 129.2 (C-2, 6), 131.0 (C-2″), 144.7 (C-β), 160.9 (C-4), 166.9 (C-2′, 4′), 192.2 (C=O).

*Papyriflavonol A (**4**)*: amorphous yellow powder; mp 202–204 °C; HRESIMS *m/z* 437.1633 $[M-H]^-$ (calcd for $C_{25}H_{25}O_7$, 437.1600); ^{1}H NMR (500 MHz, acetone–d_6) δ 1.60 (3H, s, H-4″), 1.69 (3H, s, H-5″), 1.71 (3H, s, H-4‴), 1.73 (3H, s, H-5‴), 3.31 (2H, d, J = 7.2 Hz, H-1″), 3.36 (2H, d, J = 7.5 Hz, H-1‴), 5.22 (1H, t, J =

2.5, 1.3 Hz, H-2″), 5.33 (1H, t, *J* = 2.6, 1.3 Hz, H-2‴), 6.51 (1H, s, H-8), 7.56 (1H, s, H-2′), 7.66 (1H, s, H-6′); ^{13}C NMR (125 MHz) δ 18.3 (C-4″), 18.3 (C-5‴), 22.4 (C-1‴), 26.3 (C-4‴), 26.3 (C-5″), 29.4 (C-1″), 94.2 (C-8), 104.4 (C-4a), 112.0 (C-6), 114.1 (C-2′), 121.8 (C-6′), 123.3 (C-1′), 123.6 (C-2‴), 123.8 (C-2‴), 129.5 (C-5′), 132.1 (C-3″), 133.3 (C-3‴), 137.0 (C-3), 145.4 (C-2), 146.7 (C-4′), 147.2 (C-3′), 156.0 (C-8a), 159.3 (C-5), 163.0 (C-7), 176.8 (C-4, C=O).

*3'-(3-Methylbut-2-enyl)-3',4',7-trihydroxyflavane (**5**)*: yellow sticky oil; [α]D –5.8° ($CHCl_3$, *c* 0.35); HRESIMS *m/z* 325.1448 [M-H]$^-$ (calcd for $C_{20}H_{21}O_4$, 325.1440); ^{1}H NMR (500 MHz, $CDCl_3$) δ 1.65 (3H, s, H-4″), 1.70 (3H, s, H-5″), 1.98 (1H, m, H-3a), 2.04 (2H, m, H-3b), 2.73 (2H, m, H-4), 3.41 (2H, d, *J* = 6.4 Hz, H-1″), 5.06 (1H, m, H-2), 5.13 (1H, m, H-2″), 6.36 (2H, m, H-6, 8), 6.68 (1H, m, H-5), 6.87 (2H, m, H-2′, 6′); ^{13}C NMR (125 MHz) δ 18.4 (C-4″, 5″), 25.5 (C-4), 25.6 (C-1″), 29.9 (C-3), 75.6 (C-2), 104.1 (C-8), 108.7 (C-6), 113.7 (C-6′), 114.6 (C-4a), 118.8 (C-2′), 122.9 (C-2″), 126.6 (C-3′), 130.6 (C-5), 132.5 (C-1′), 133.5 (C-3″), 142.8 (C-4′), 143.8 (C-5′), 155.3 (C-7), 156.5 (C-8a).

*Kazinol A (**6**)*: yellowish powder; mp 129–130 °C; [α]D –10.7° ($CHCl_3$, *c* 0.13); HRESIMS *m/z* 393.2055 [M-H]$^-$ (calcd for $C_{25}H_{29}O_4$, 393.2066); ^{1}H NMR (500 MHz, acetone–d_6) δ 1.67 (6H, s, H-4‴, 5‴), 1.71 (3H, s, H-4″), 1.76 (3H, s, H-5″), 1.88 (1H, m, H-3), 2.07 (1H, m, H-3), 2.71 (1H, m, H-4), 2.82 (1H, m, H-4), 3.34 (2H, m, H-1‴), 3.42 (2H, d, *J* = 10.5 Hz, H-1″), 5.04 (1H, d, *J* = 1.8 Hz, H-2), 5.07 (1H, t, *J* = 17.4 Hz, *J* = 3.2 Hz, H-2‴), 5.12 (1H, t, *J* = 10.9 Hz, *J* = 2.2 Hz, 2″), 6.27 (1H, d, *J* = 2.4 Hz, H-8), 6.37 (1H, d, *J* = 2.4 Hz, *J* = 8.2, H-6), 6.88 (1H, s, H-6′), 6.90 (1H, d, *J* = 6.2 Hz, H-5); ^{13}C NMR (125 MHz) δ 17.1 (C-4‴), 17.2 (C-4″), 24.8 (C-5‴), 24.9(C-5″), 25.2 (C-3), 25.2 (C-1″), 26.8 (C-4), 26.8 (C-1″), 74.7 (C-2), 103.0 (C-8), 107.9 (C-6), 110.2 (C-4a), 110.8 (C-6′), 112.9 (C-2″), 123.7 (C-2‴), 124.6 (C-5′), 126.7 (C-2′), 129.0 (C-5), 130.1 (C-1′), 130.2 (C-3‴), 131.1 (C-3″), 142.4 (C-4′), 142.9 (C-3′), 156.5 (C-7), 156.6 (C-8a).

Kazinol B (7): amorphous yellow powder; mp 86–88 °C; [α]D –19.0° ($CHCl_3$, *c* 0.38); HRESIMS *m/z* 391.1912 [M-H]$^-$ (calcd for $C_{25}H_{27}O_4$, 391.1909); ^{1}H NMR (500 MHz, acetone–d_6) δ 1.34 (3H, s, H-5‴), 1.37 (3H, s, H-4‴), 1.58 (3H, s, H-5″), 1.62 (3H, s, H-4″), 1.89 (1H, m, H-3), 1.99 (1H, m, H-3), 2.64 (1H, m, H-4), 2.78 (1H, m, H-4), 3.33 (2H, t, *J* = 14.8 Hz, *J* = 7.7 Hz, H-1″), 4.99 (2H, dd, J_1 = 10.7 Hz, J_2 = 1.9 Hz, H-2), 5.06 (2H, t, *J* = 12.1 Hz, *J* = 6.3 Hz, H-2″), 5.46 (1H, d, *J* = 9.8 Hz, H-2‴), 6.20 (1H, d, *J* = 9.9 Hz, H-

1‴), 6.27 (1H, s, H-8), 6.29 (1H, d, J = 2.6 Hz, H-6), 6.65 (1H, s, H-2′), 6.82 (1H, d, J = 8.4 Hz, H-5); ^{13}C NMR (125 MHz) δ 14.5 (C-4″), 18.3 (C-3), 19.5 (C-1″), 26.1 (C-5″), 28.2 (C-5‴), 28.5 (C-4‴), 30.1 (C-4), 74.5 (C-2), 77.7 (C-3‴), 104.1 (C-8), 108.4 (C-6), 114.5 (C-4a), 115.5 (C-1‴), 119.4 (C-5′), 122.7 (C-6′), 123.3 (C-2″), 126.2 (C-2′), 130.3 (C-5), 130.5 (C-2‴), 132.0 (C-1′), 132.7 (C-3″), 139.0 (C-4′), 142.6 (C-3′), 155.4 (C-7), 156.8 (C-8a).

*Broussoflavan A (**8**)*: yellowish powder; mp 238–240 °C; $[\alpha]_D$ –17.65° (acetone, *c* 0.092); HRESIMS *m/z* 425.1955 $[M-H]^-$ (calcd for $C_{25}H_{29}O_6$, 425.1964); ^{1}H NMR (500 MHz, acetone-d_6) δ 1.23 (3H, s, H-4″), 1.48 (3H, s, H-5‴), 1.65 (3H, s, H-4″), 1.72 (3H, s, H-5″), 1.96 (1H, m, H-3), 2.10 (1H, m H-3), 2.73 (1H, m ,H-4), 2.91 (1H, m ,H-4), 3.46 (1H, m, H-1″), 3.59 (1H, d, J = 8.5 Hz, H-1‴), 4.54 (1H, d, J = 8.5 Hz, H-2‴), 5.10 (1H, d, J = 9.0 Hz, H-2), 5.18 (1H, brt, H-2″), 6.31 (1H, s, H-8), 6.39 (1H, d, J = 8.5 Hz, H-6), 6.91 (1H, d, J = 8.5 Hz, H-5), 7.35 (1H, s, H-6′); ^{13}C NMR (125 MHz) δ 18.5 (C-4″), 20.2 (C-4‴), 25.6 (C-1″), 26.3 (C-5″), 26.4 (C-4), 27.4 (C-5‴), 31.1 (C-3), 70.3 (C-2‴), 76.2 (C-2), 77.3 (C-1‴), 80.4 (C-3‴), 104.4 (C-8), 109.3 (C-6), 114.2 (C-4a), 117.0 (C-6′), 123.8 (C-5′), 125.0 (C-2″), 125.6 (C-2′), 131.3 (C-5), 131.4 (C-3″), 133.4 (C-1′), 140.4 (C-4′), 143.9 (C-3′), 157.7 (C-7), 157.9 (C-8a).

Kazinol F (***9***). Crystallized from benzene to give colorless needles, mp 108-109 °C; HRESIMS *m/z* 395.2201 $[M-H]^-$ (calcd for $C_{25}H_{31}O_4$, 395.2222); ^{1}H NMR (500 MHz, acetone–d_6) δ 1.51 (3H, s, CH_3-10″), 1.52 (3H, s, CH_3-11″), 1.57 (3H, s, CH_3-16″), 1.59 (3H, s, CH_3-15″), 1.64 (2H, m, H-2), 2.34 (2H, m, H-3), 2.45 (2H, m, H-1), 3.08 (2H, d, J = 5.8 Hz, H-12″), 3.21 (2H, d, J = 6.5 Hz, H-7″), 4.82 (1H, m, H-13″), 4.96 (1H, m, H-8″), 6.12 (1H, dd, J = 8.1, 2.3 Hz, H-5′), 6.24 (1H, d, J = 2.3 Hz, H-3′), 6.42 (1H, s, H-2″), 6.74 (1H, d, J = 8.1 Hz, H-6′). ^{13}C NMR (125 MHz, acetone–d_6) δ 34.0 (C-1), 31.0 (C-2), 33.4 (C-3), 120.9 (C-1′), 157.0 (C-2′), 103.8 (C-3′), 157.7 (C-4′), 107.6 (C-5′), 131.5 (C-6′), 40.2 (C-7′), 133.0 (C-1″), 114.9 (C-2″), 143.3 (C-3″), 142.5 (C-4″), 130.8 (C-5″), 128.1 (C-6″), 26.6 (C-7″), 125.3 (C-8″), 131.1 (C-9″), 18.5 (C-10″), 26.2 (C-11″), 28.5 (C-12″), 126.1 (C-13″), 131.1 (C-14″), 18.4 (C-15″), 26.2 (C-16″).

Kazinol J (**10**) Crystallized from benzene to give colorless needles, mp 117-119 °C; HRESIMS *m/z* 409.23705 $[M-H]^-$ (calcd for $C_{26}H_{33}O_4$, 409.2379); ^{1}H NMR (500 MHz, acetone–d_6) δ 1.57 (3H, s, CH_3-10″), 1.61 (3H, s, CH_3-11″), 1.62 (3H, s, CH_3-16″), 1.69 (3H, s, CH_3-15″), 1.67 (2H, m, H-2), 2.41 (2H, m,

H-3), 2.46 (2H, m, H-1), 3.10 (2H, d, J = 5.5 Hz, H-12″), 3.2 (2H, d, J = 6.3 Hz, H-7″), 3.63 (3H, s, OCH_3), 4.86 (1H, m, H-13″), 5.04 (1H, m, H-8″), 6.22 (1H, dd, J = 8.1, 2.3 Hz, H-5′), 6.23 (1H, d, J = 2.3 Hz, H-3′), 6.50 (1H, s, H-2″), 6.83 (1H, d, J = 8.1 Hz, H-6′). ^{13}C NMR (125 MHz, acetone–d_6) δ 33.2 (C-1), 29.8 (C-2), 31.8 (C-3), 122.7 (C-1′), 158.6 (C-2′), 99.4 (C-3′), 155.0 (C-4′), 107.0 (C-5′), 131.0 (C-6′), 133.8 (C-1″), 114.5 (C-2″), 141.9 (C-3″), 140.5 (C-4″), 130.2 (C-5″), 126.9 (C-6″), 27.9 (C-7″), 123.2 (C-8″), 130.5 (C-9″), 25.9 (C-10″), 18.1 (C-11″), 26.3 (C-12″), 124.4 (C-13″), 133.6 (C-14″), 25.8 (C-15″), 18.1 (C-16″), 55.7 (2′-OCH_3).

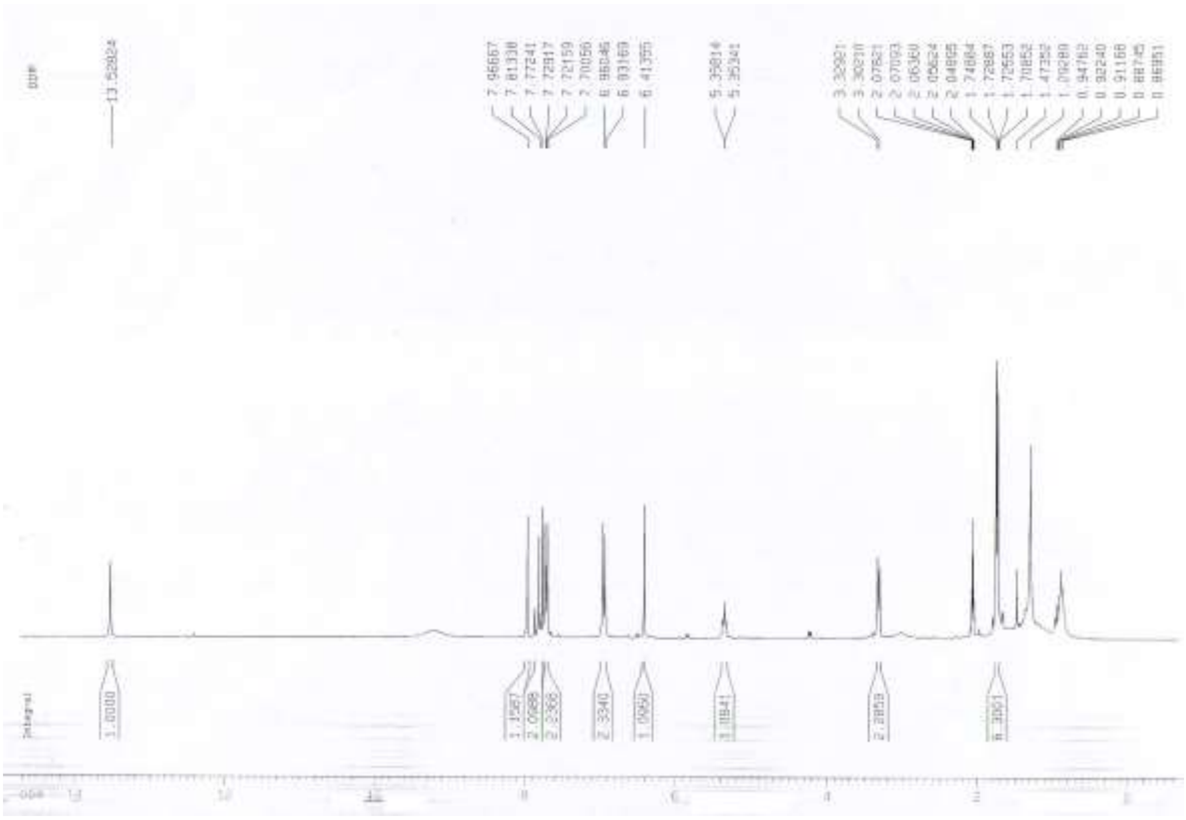

Figure S1. ^{1}H-NMR spectrum of compound **1** (500 MHz, acetone-d_6).

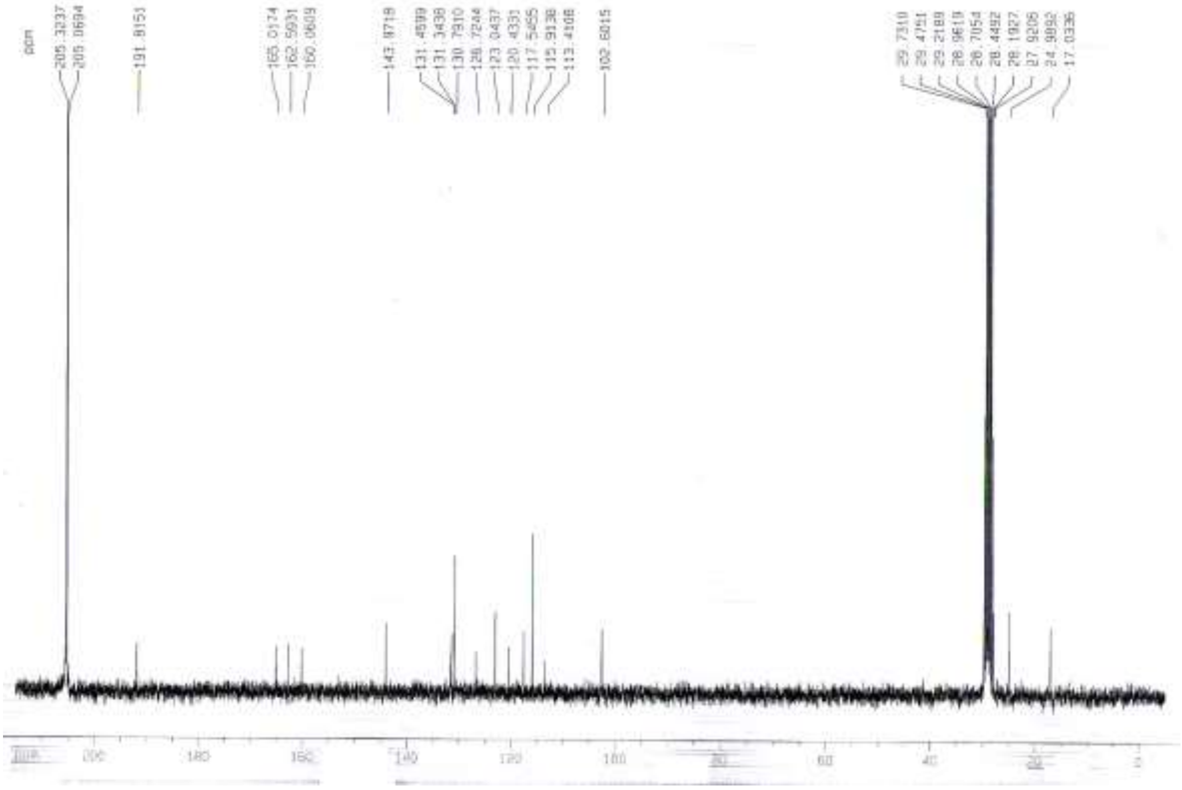

Figure S2. ^{13}C-NMR spectrum of compound **1** (125 MHz, acetone-d_6).

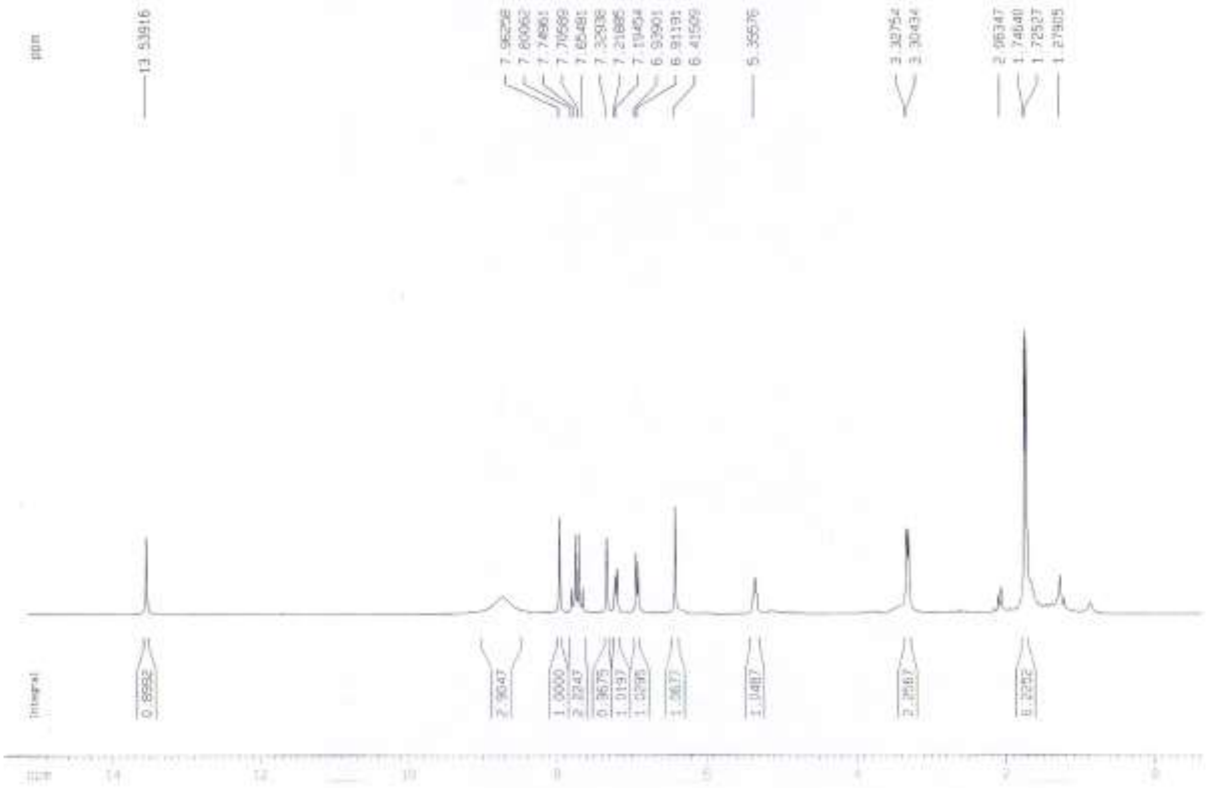

Figure S3. ^{1}H-NMR spectrum of compound **2** (500 MHz, acetond-d_6).

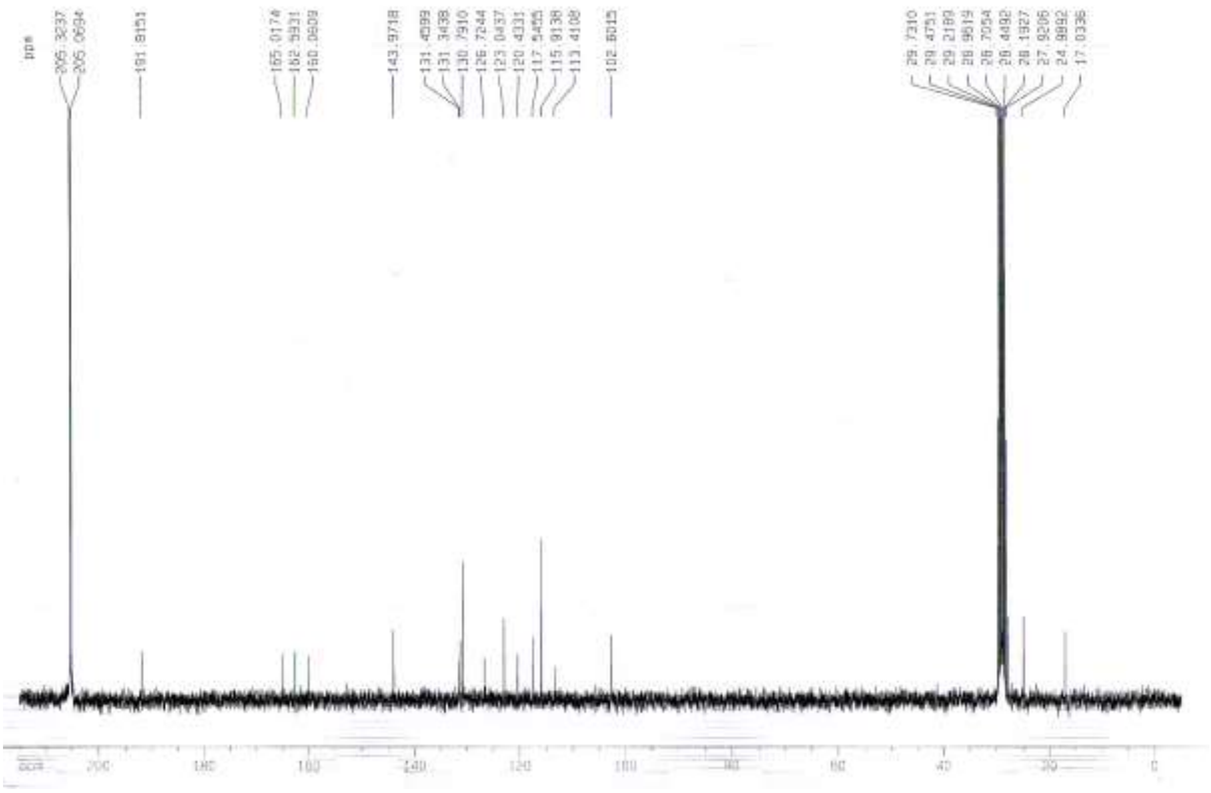

Figure S4. ^{13}C-NMR spectrum of compound **2** (125 MHz, acetond-d_6).

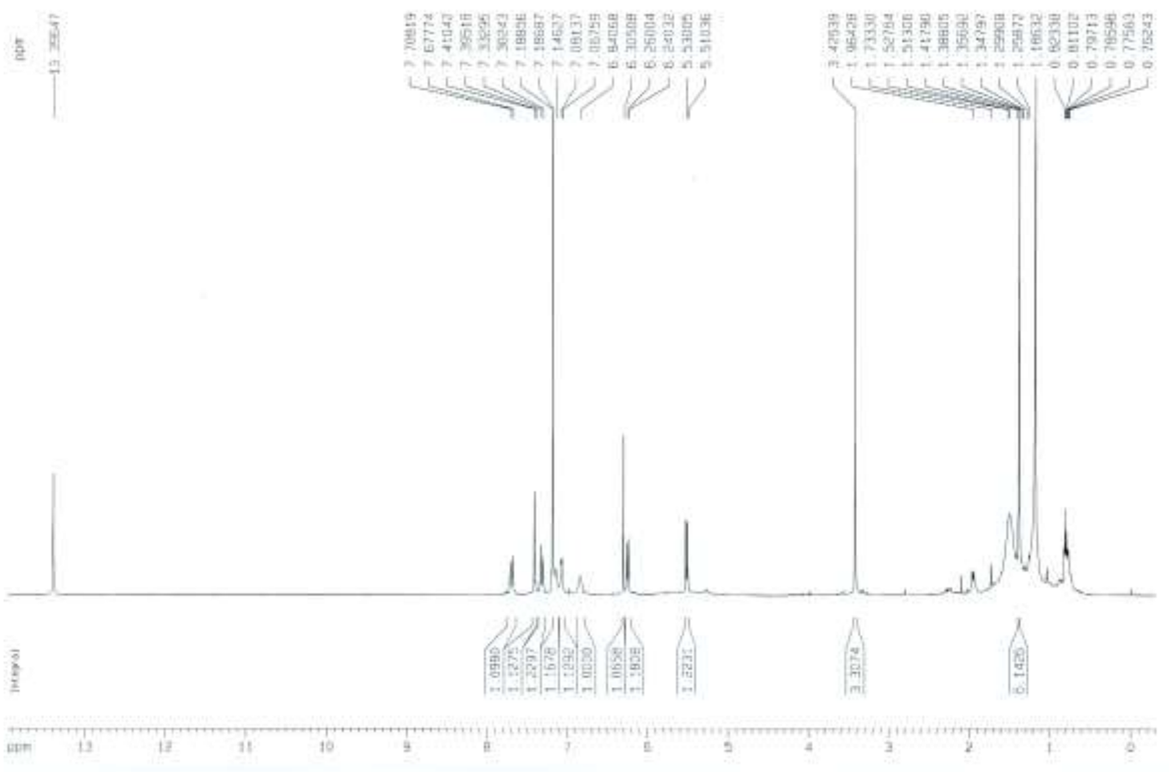

Figure S5. ^{1}H-NMR spectrum of compound **3** (500 MHz, $CDCl_3$).

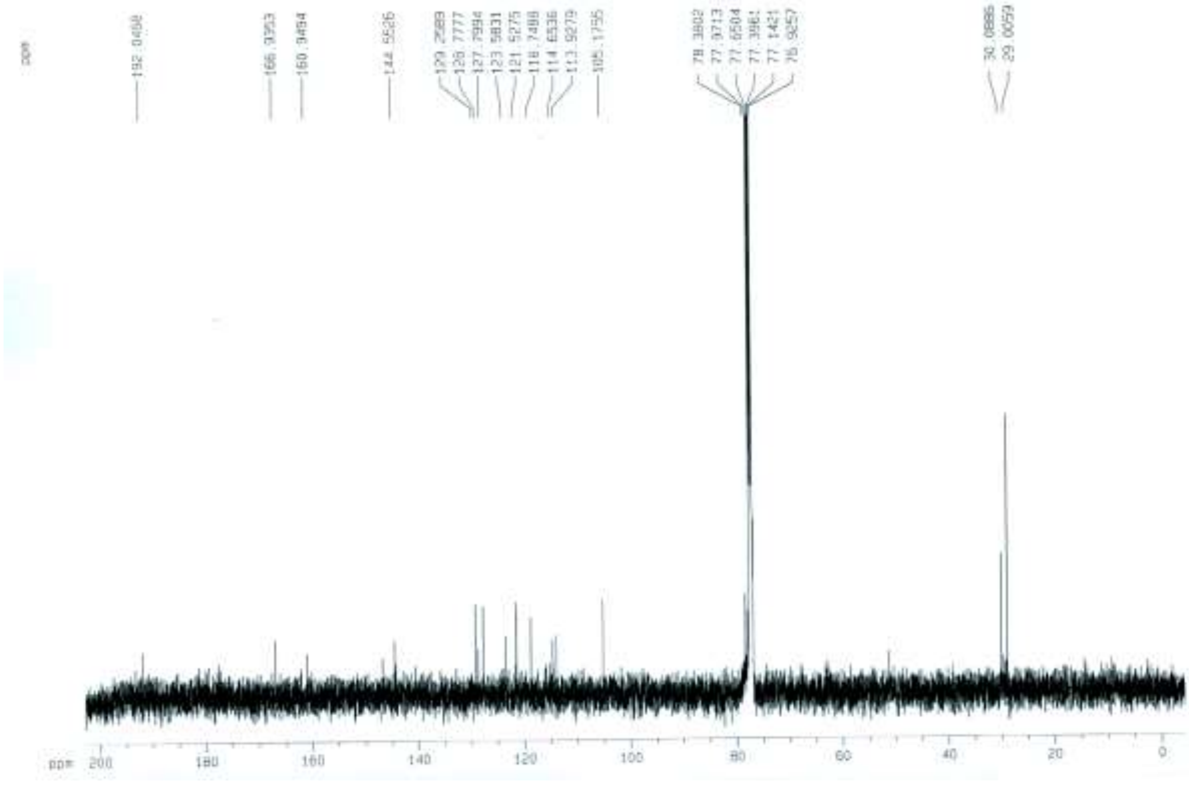

Figure S6. ^{1}H-NMR spectrum of compound **3** (125 MHz, $CDCl_3$).

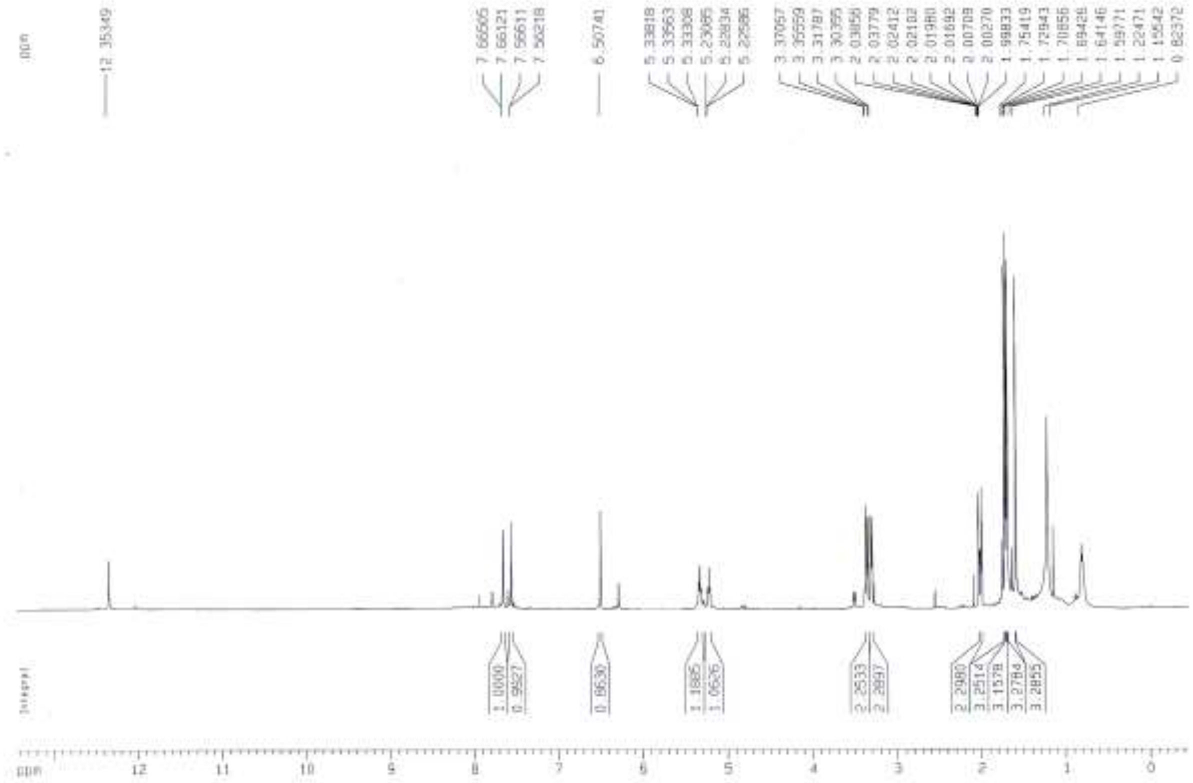

Figure S7. ^{1}H-NMR spectrum of compound **4** (500 MHz, CD_3OD).

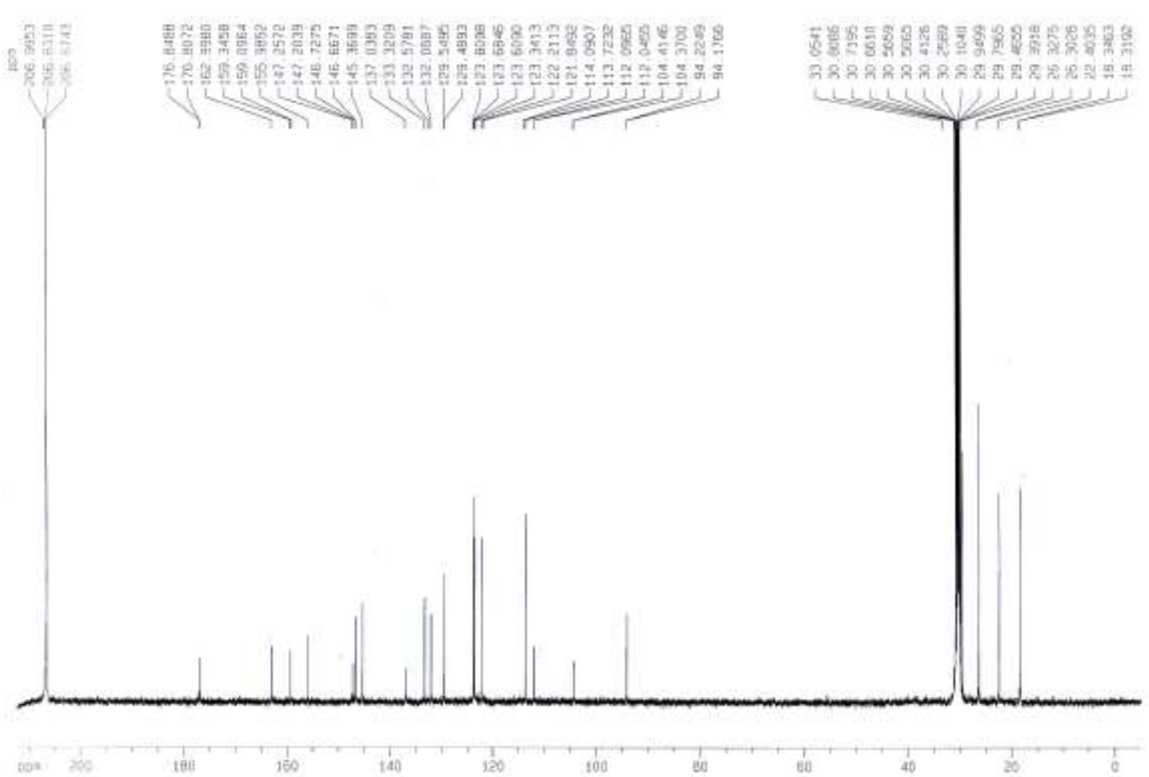

Figure S8. ^{13}C-NMR spectrum of compound **4** (125 MHz, CD_3OD).

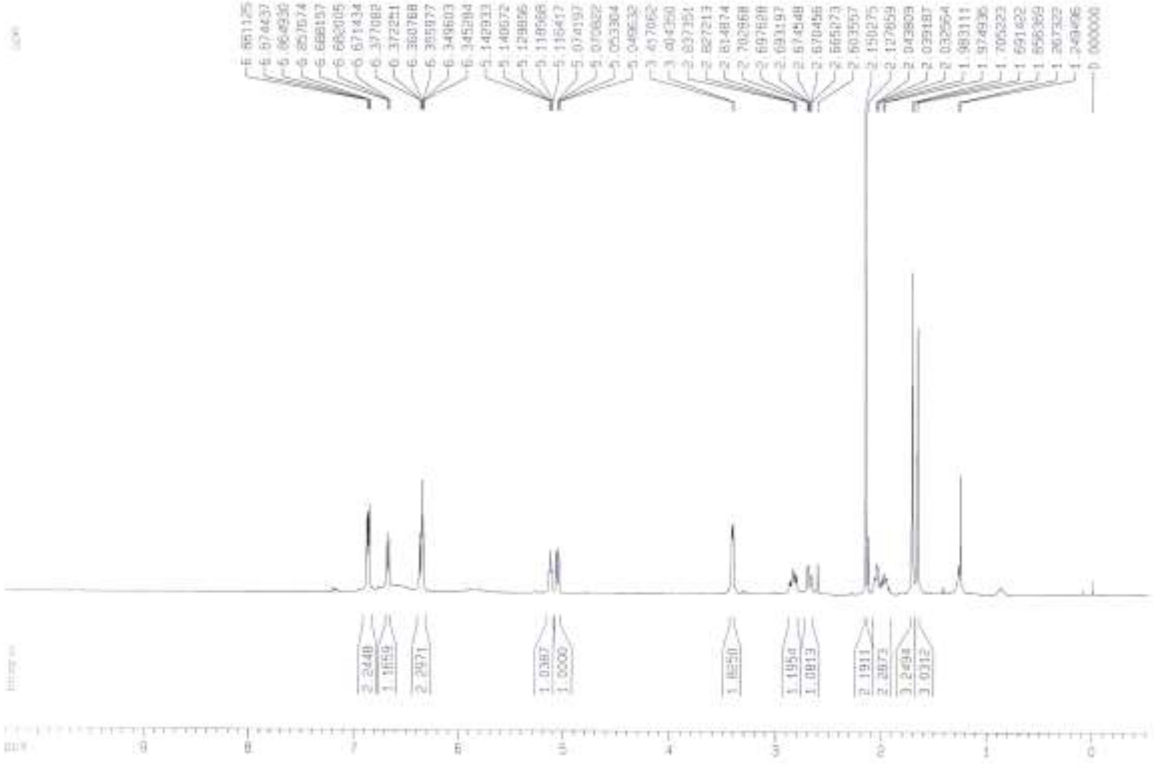

Figure S9. ^{1}H-NMR spectrum of compound **5** (500 MHz, $CDCl_3$).

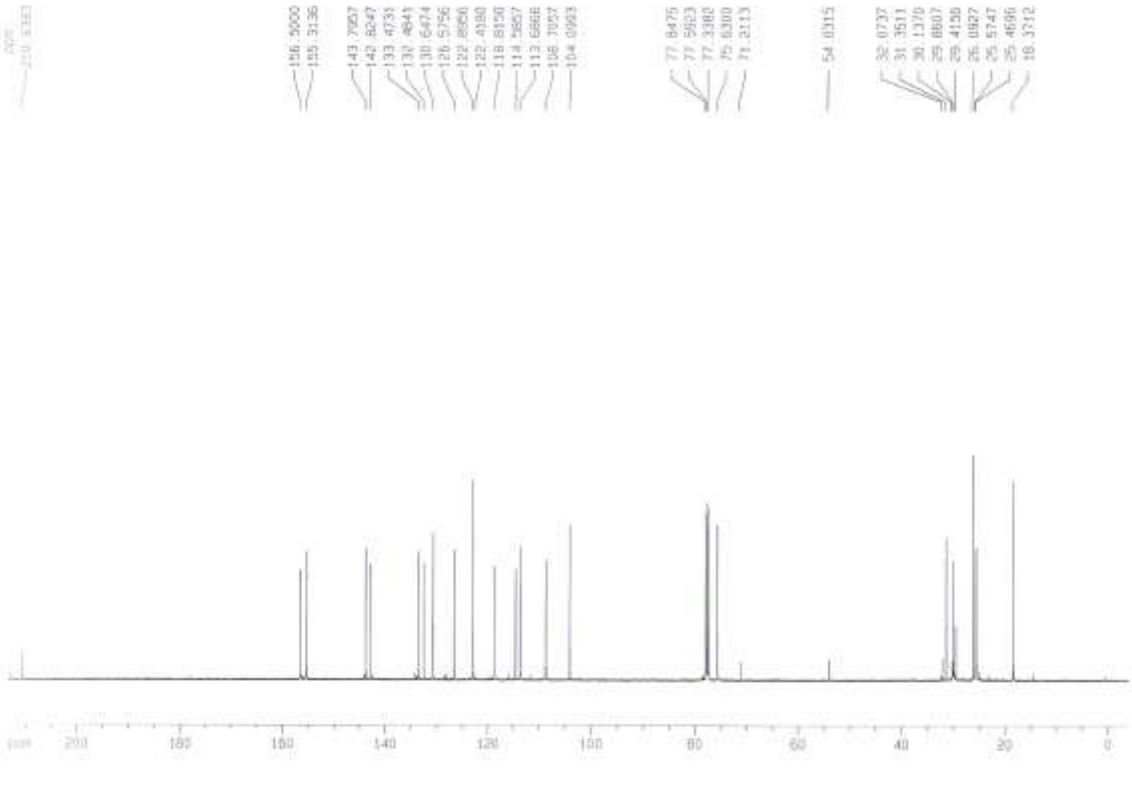

Figure S10. ^{13}C-NMR spectrum of compound **5** (125 MHz, $CDCl_3$).

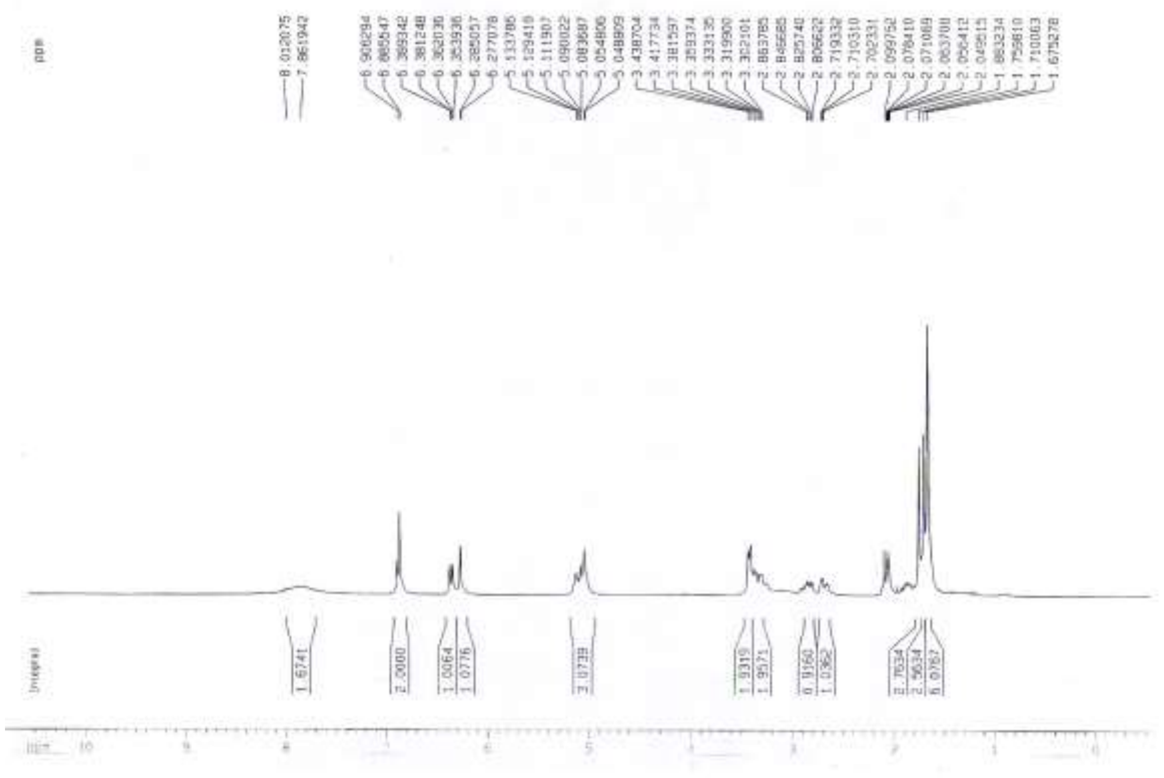

Figure S11. ^{1}H-NMR spectrum of compound **6** (500 MHz, acetone-d_6).

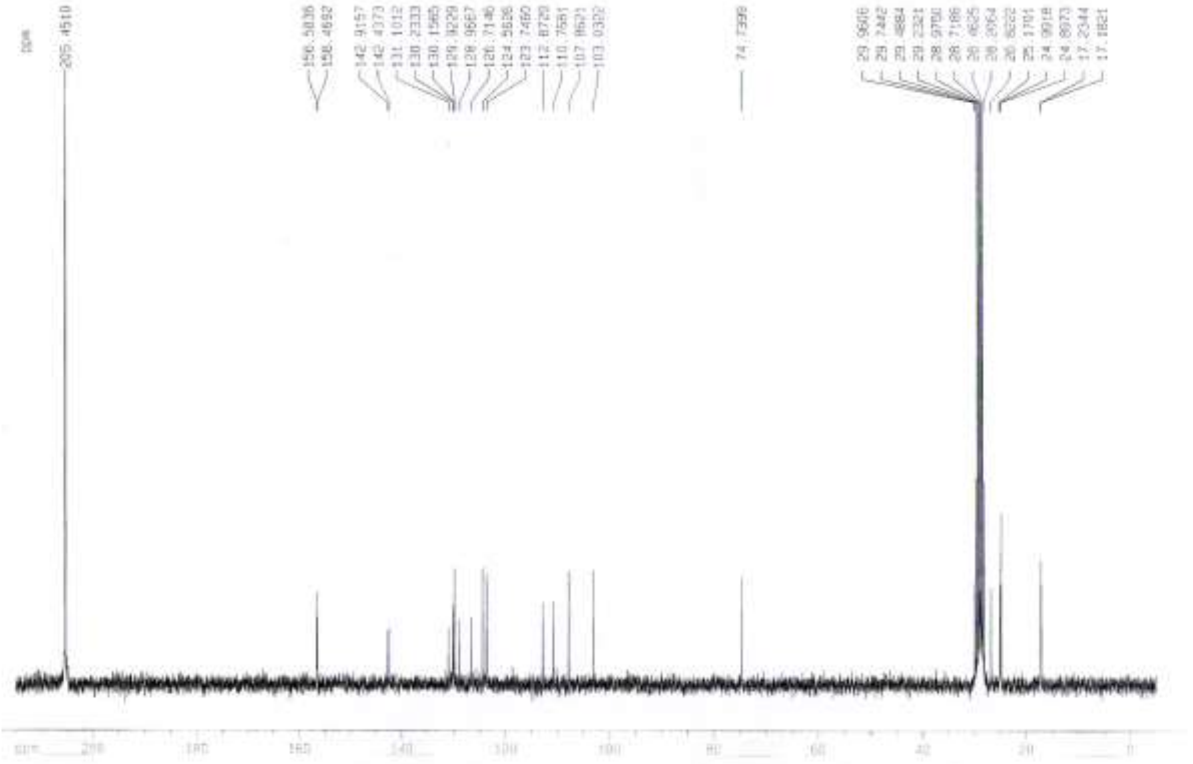

Figure S12. ^{13}C-NMR spectrum of compound **6** (125 MHz, acetone-d_6).

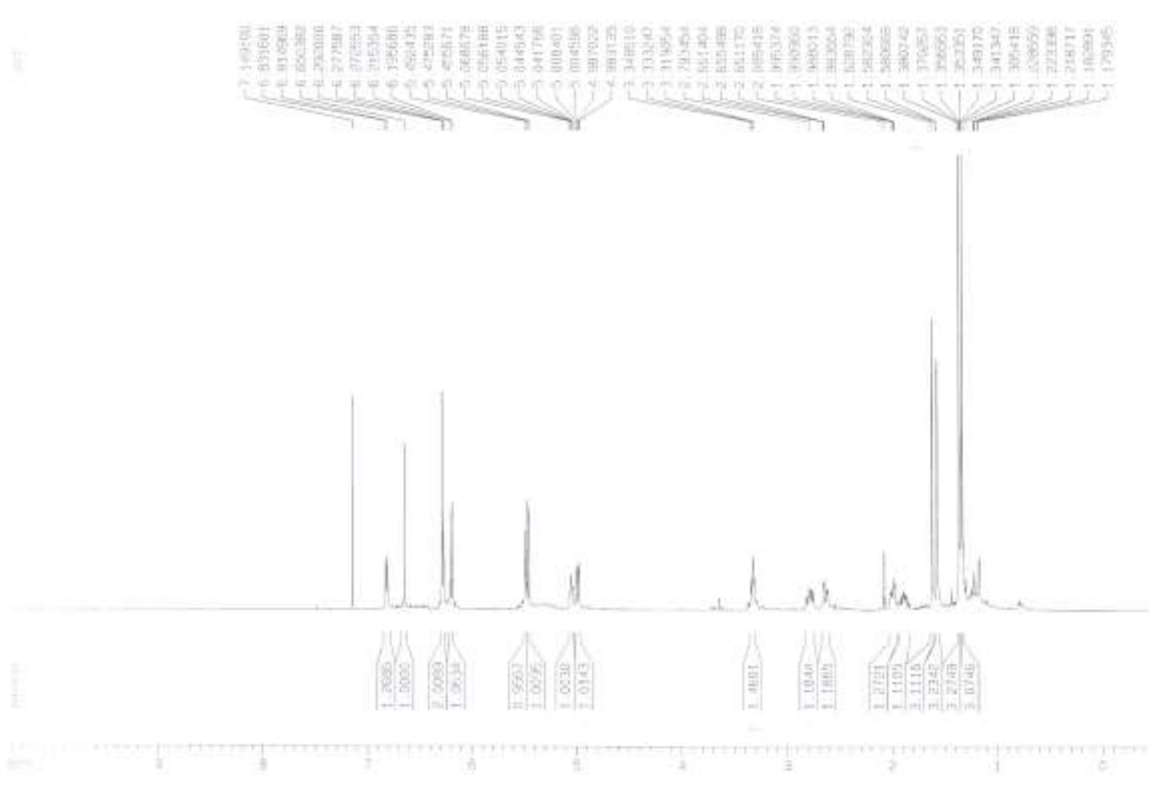

Figure S13. ^{1}H-NMR spectrum of compound **7** (500 MHz, $CDCl_3$).

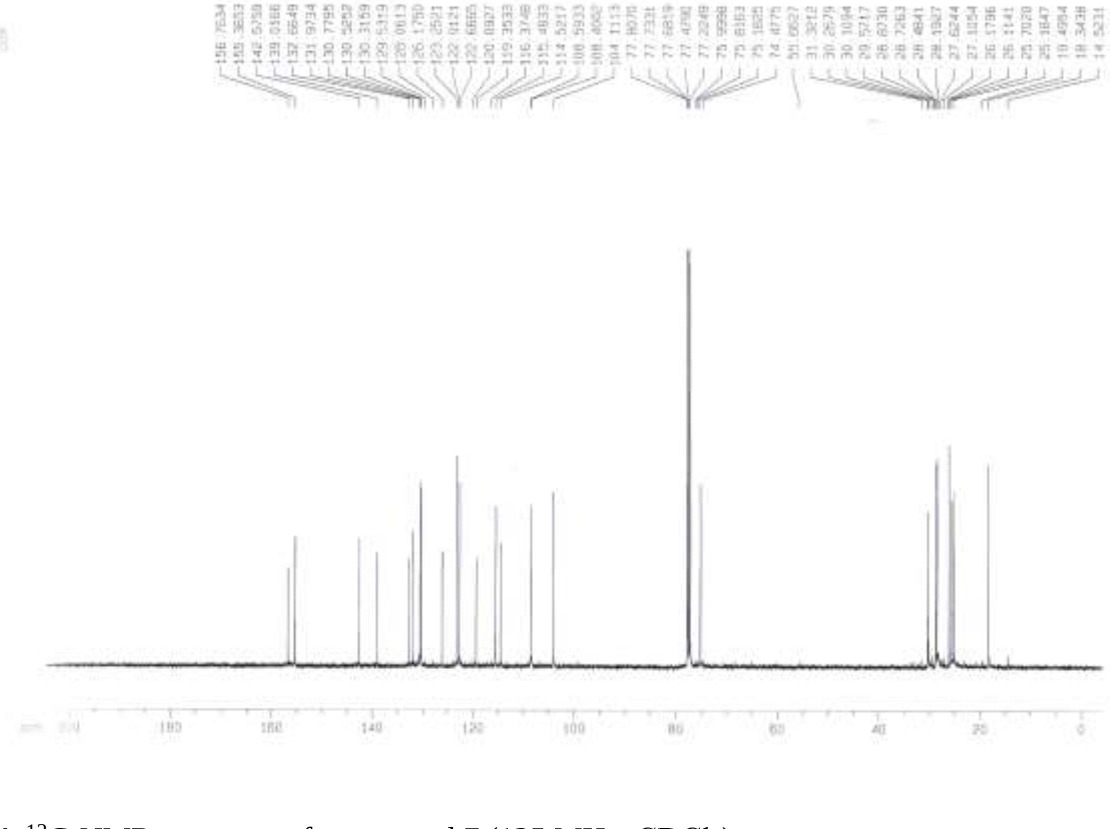

Figure S14. ^{13}C-NMR spectrum of compound **7** (125 MHz, $CDCl_3$).

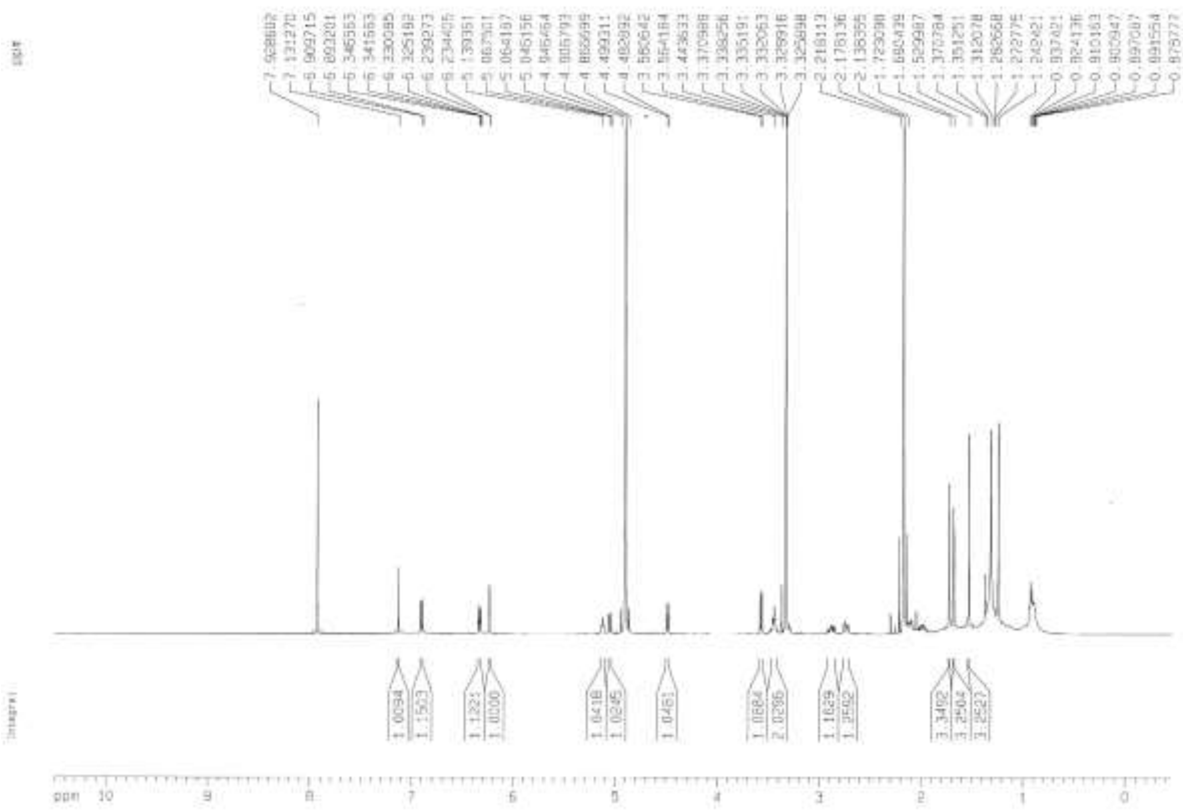

Figure S15. ^{1}H-NMR spectrum of compound **8** (500 MHz, CD_3OD).

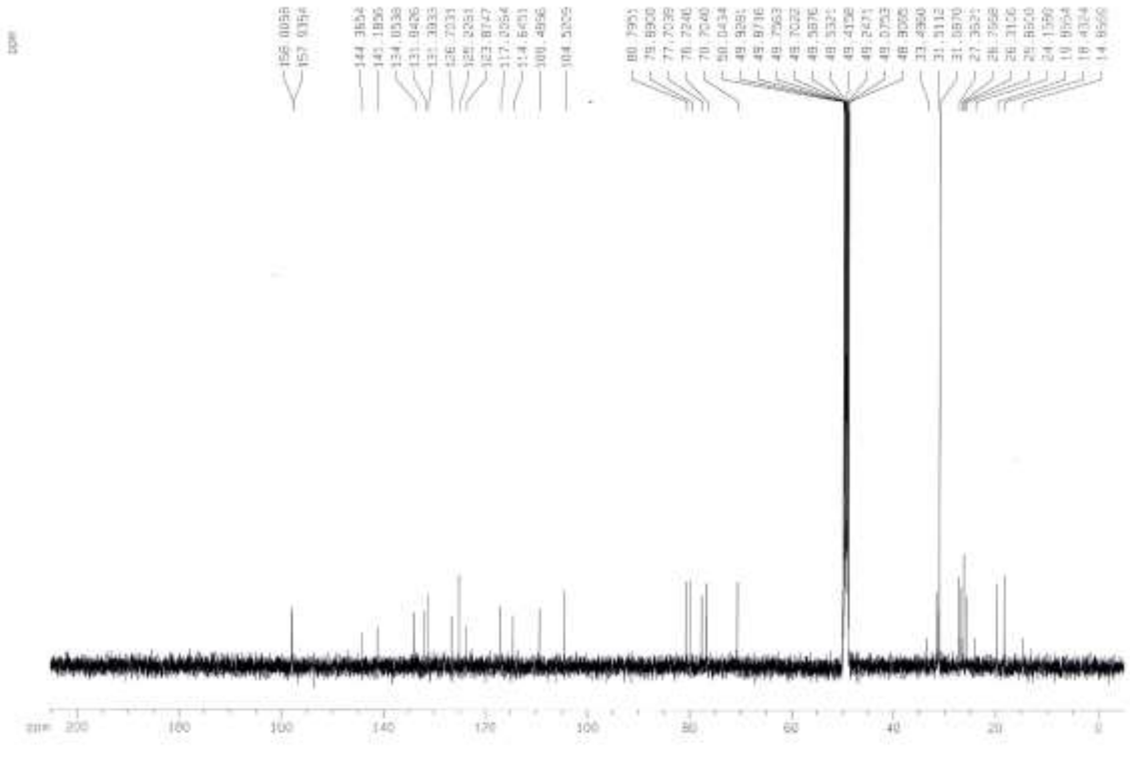

Figure S16. ^{13}C-NMR spectrum of compound **8** (125 MHz, CD_3OD).

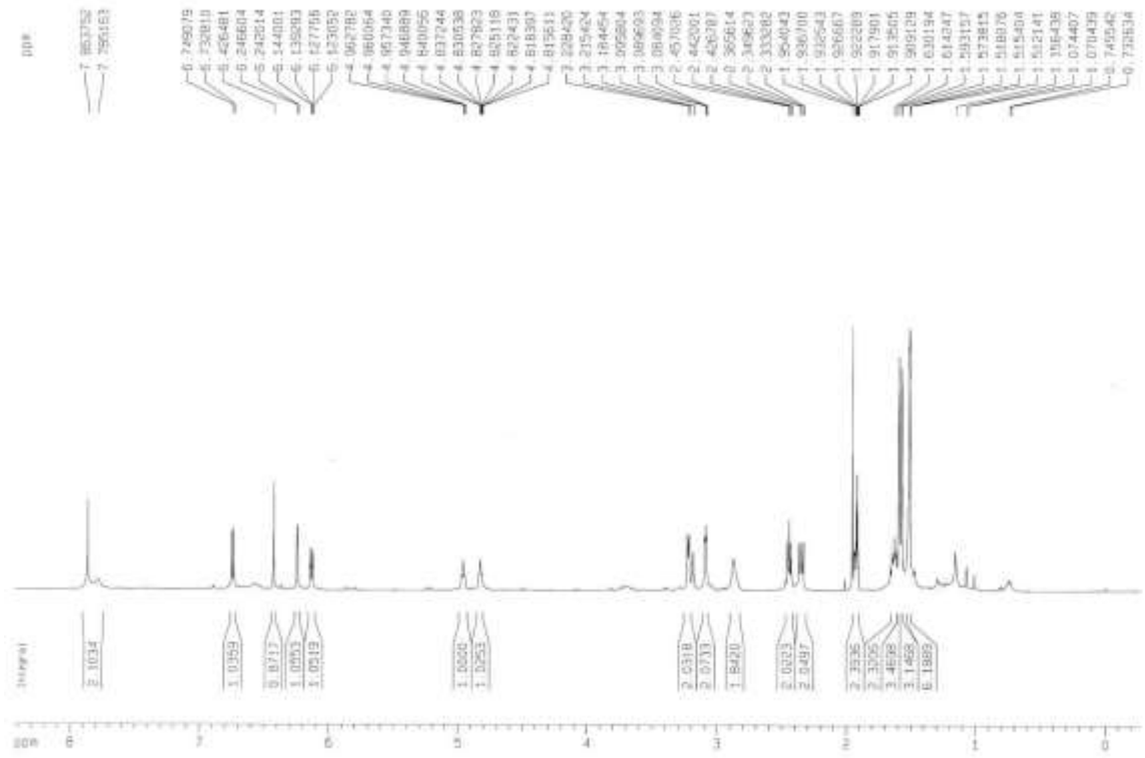

Figure S17. ^{1}H-NMR spectrum of compound **9** (500 MHz, acetone-d_6).

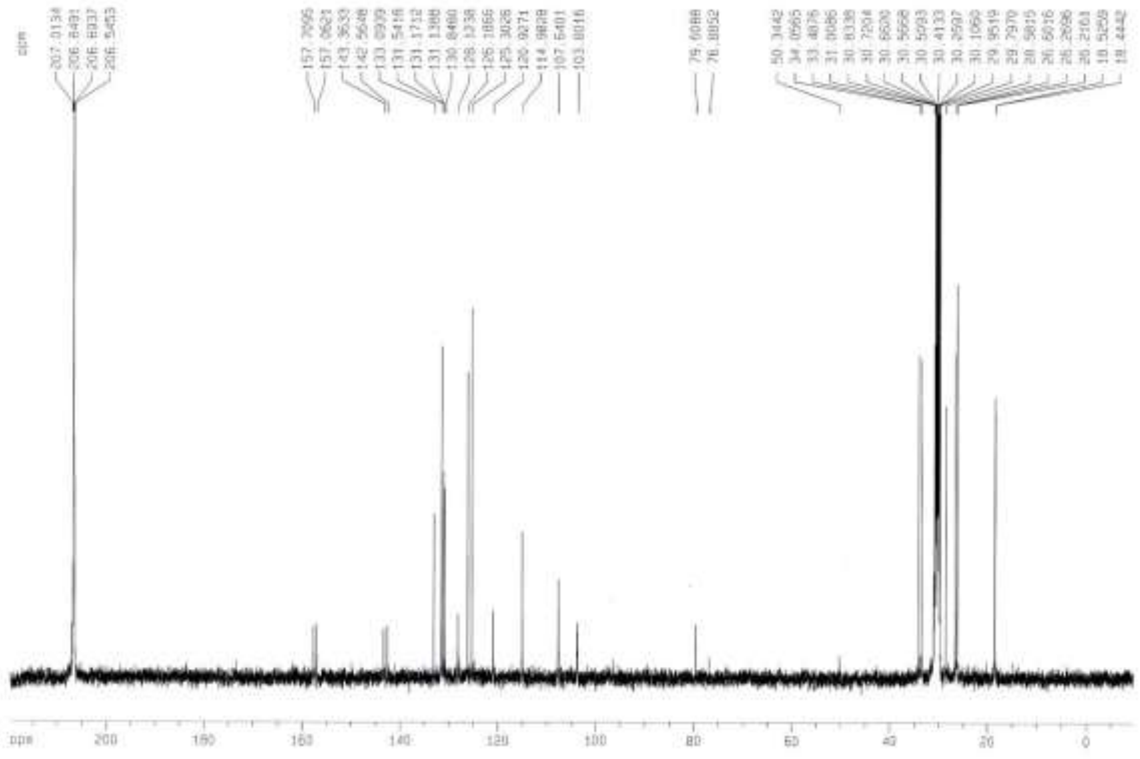

Figure S18. ^{13}C-NMR spectrum of compound **9** (125 MHz, acetone-d_6).

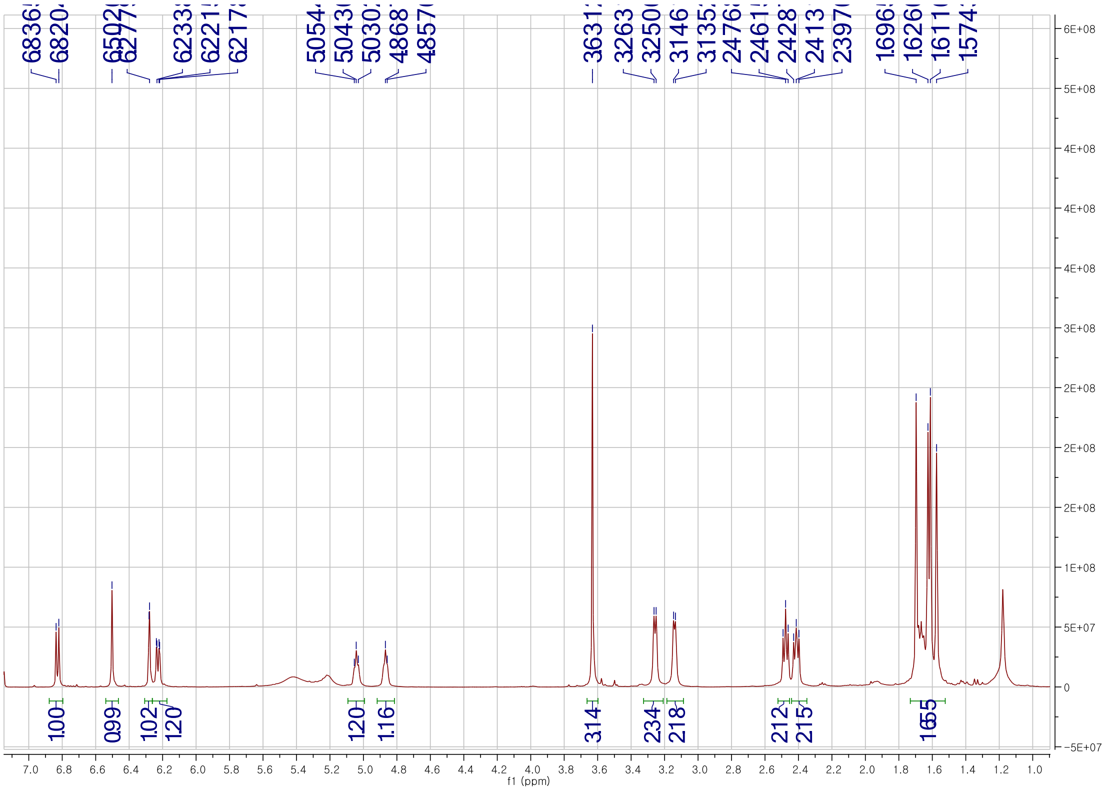

Figure S19. ^{1}H-NMR spectrum of compound **10** (500 MHz, $CDCl_3$).

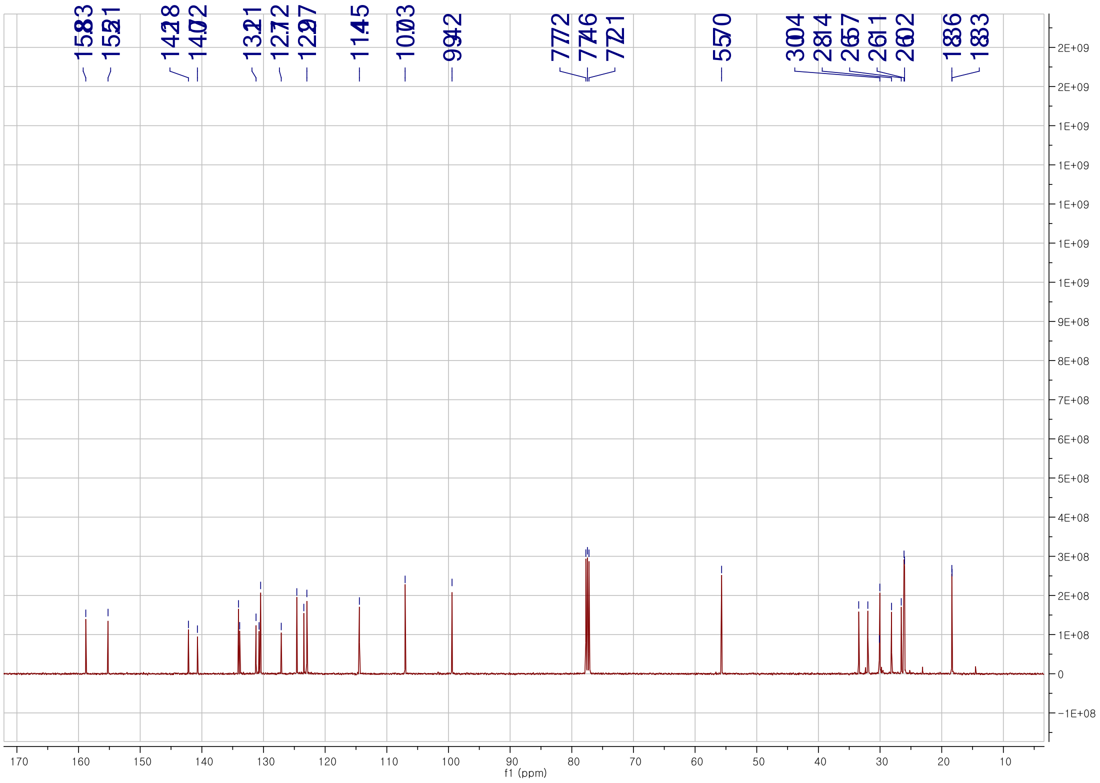

Figure S20. ^{13}C-NMR spectrum of compound **10** (125MHz, $CDCl_3$).

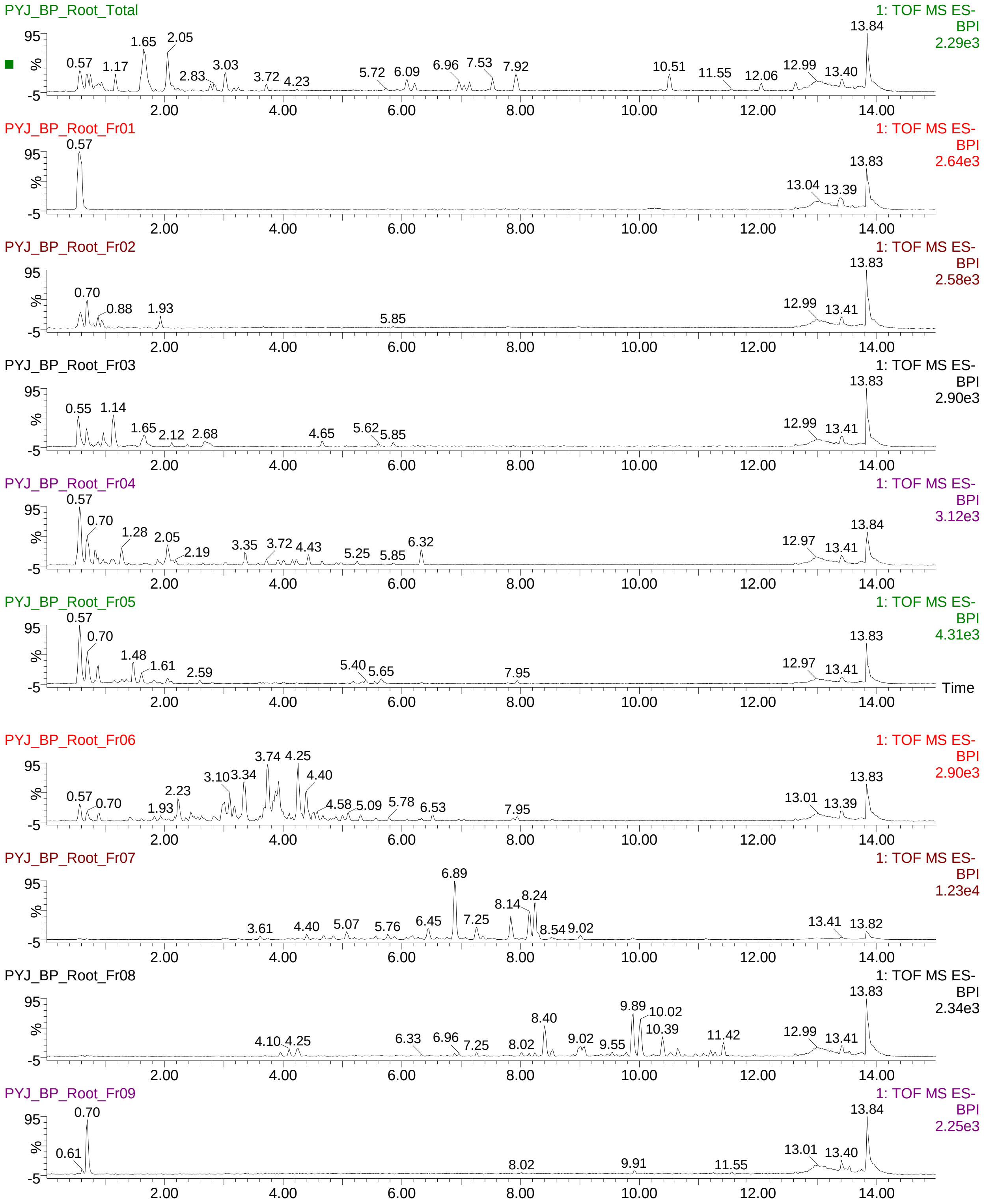

Figure S21. UPLC-QTof-MS chromatogram of separated fractions (BP Fr.1-9) using MPLC from roots extract of *B. papyrifera*.

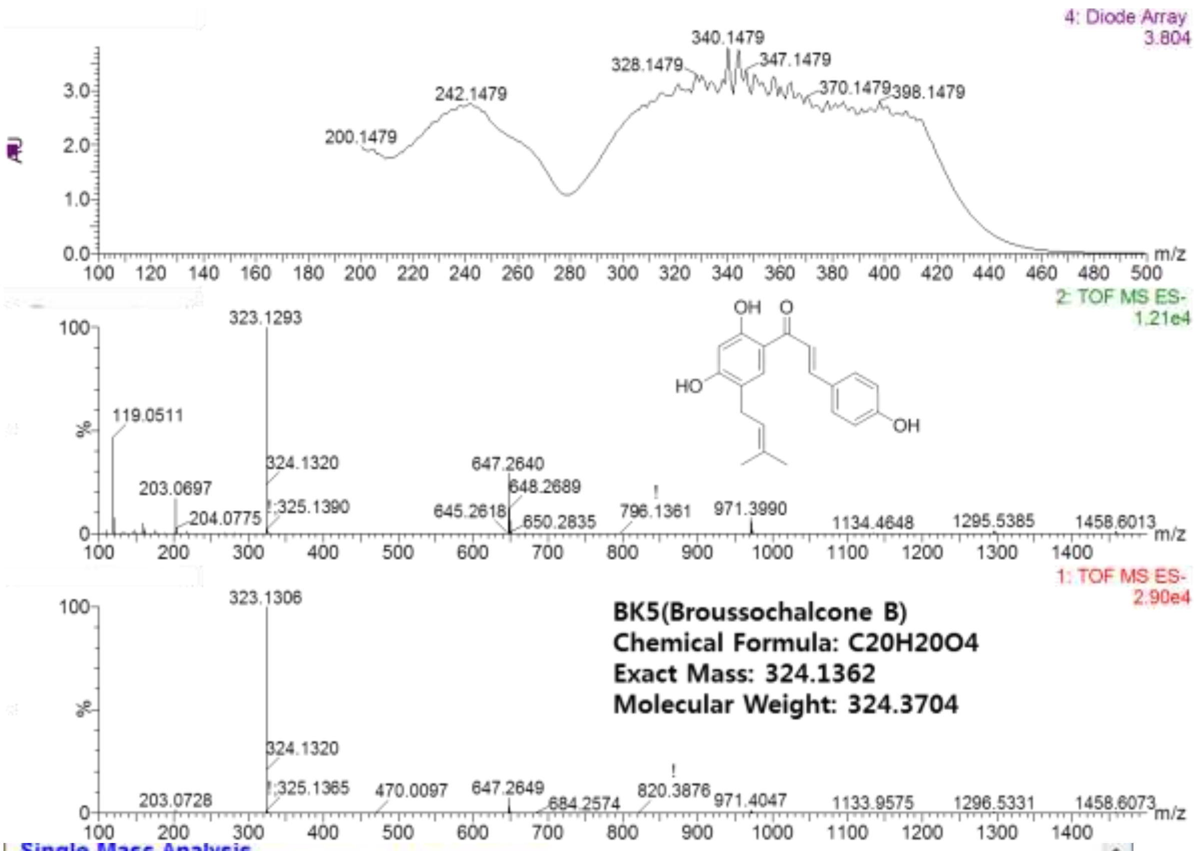

Single Mass Analysis

Tolerance = 10.0 PPM / DBE: min = -1.5, max = 50.0

Element prediction: Off

Number of isotope peaks used for i-FIT = 3

Monoisotopic Mass, Even Electron Ions

64 formula(e) evaluated with 1 results within limits (up to 50 best isotopic matches for each mass)

Elements Used:

Mass	Calc. Mass	mDa	PPM	DBE	Formula	i-FIT	i-FIT Norm	Fit Conf %	C	H	O
323.1306	323.1283	2.3	7.1	11.5	C20 H19 O4	216.7	n/a	n/a	20	19	4

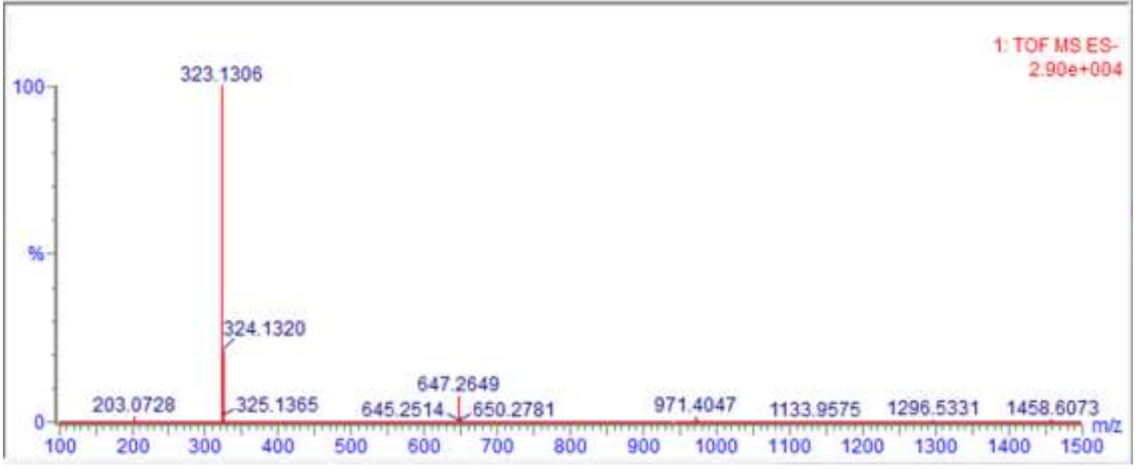

Figure S22. UPLC-QTOF-MS and HREIMS data of compound **1**.

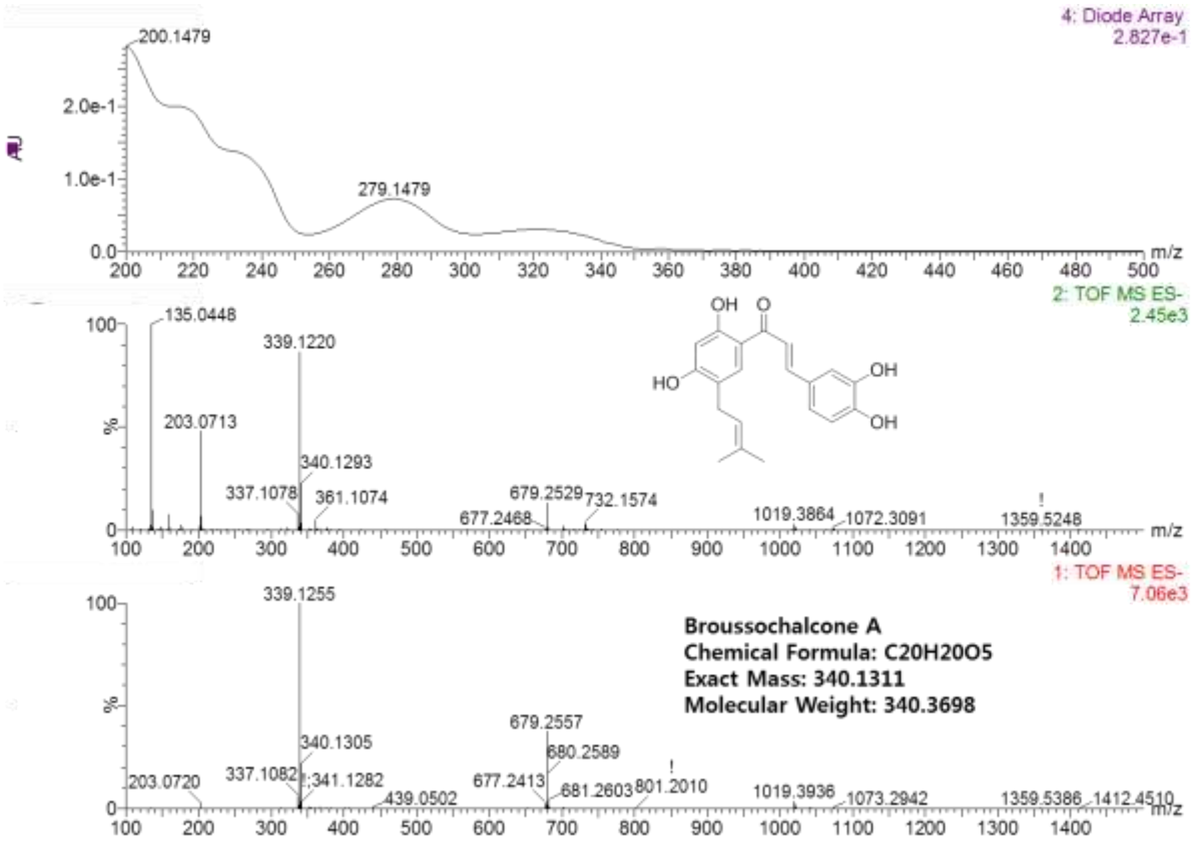

Single Mass Analysis

Tolerance = 10.0 PPM / DBE: min = -1.5, max = 50.0

Element prediction: Off

Number of isotope peaks used for i-FIT = 3

Monoisotopic Mass, Even Electron Ions

69 formula(e) evaluated with 1 results within limits (up to 50 best isotopic matches for each mass)

Elements Used:

Mass	Calc. Mass	mDa	PPM	DBE	Formula	i-FIT	i-FIT Norm	Fit Conf %	C	H	O
339.1255	339.1232	2.3	6.8	11.5	C20 H19 O5	63.5	n/a	n/a	20	19	5

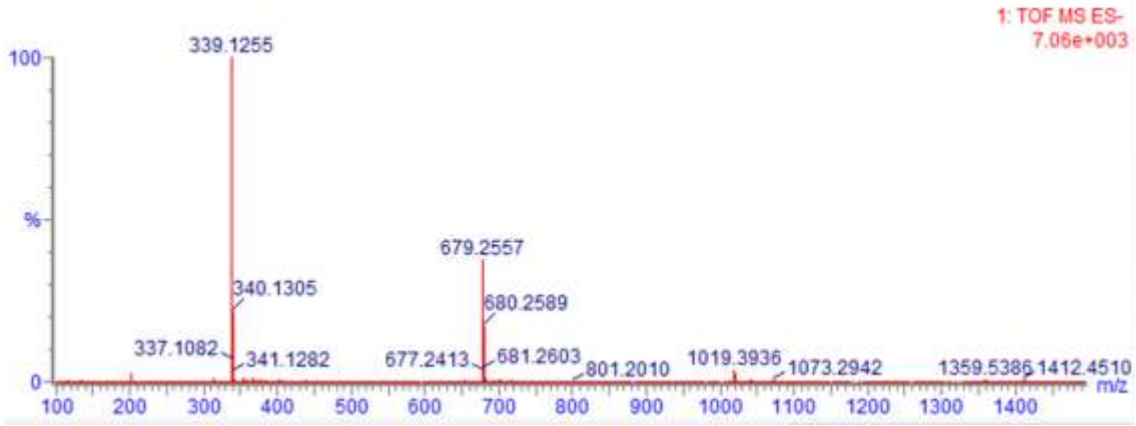

Figure S23. UPLC-QTOF-MS and HREIMS data of compound **2**.

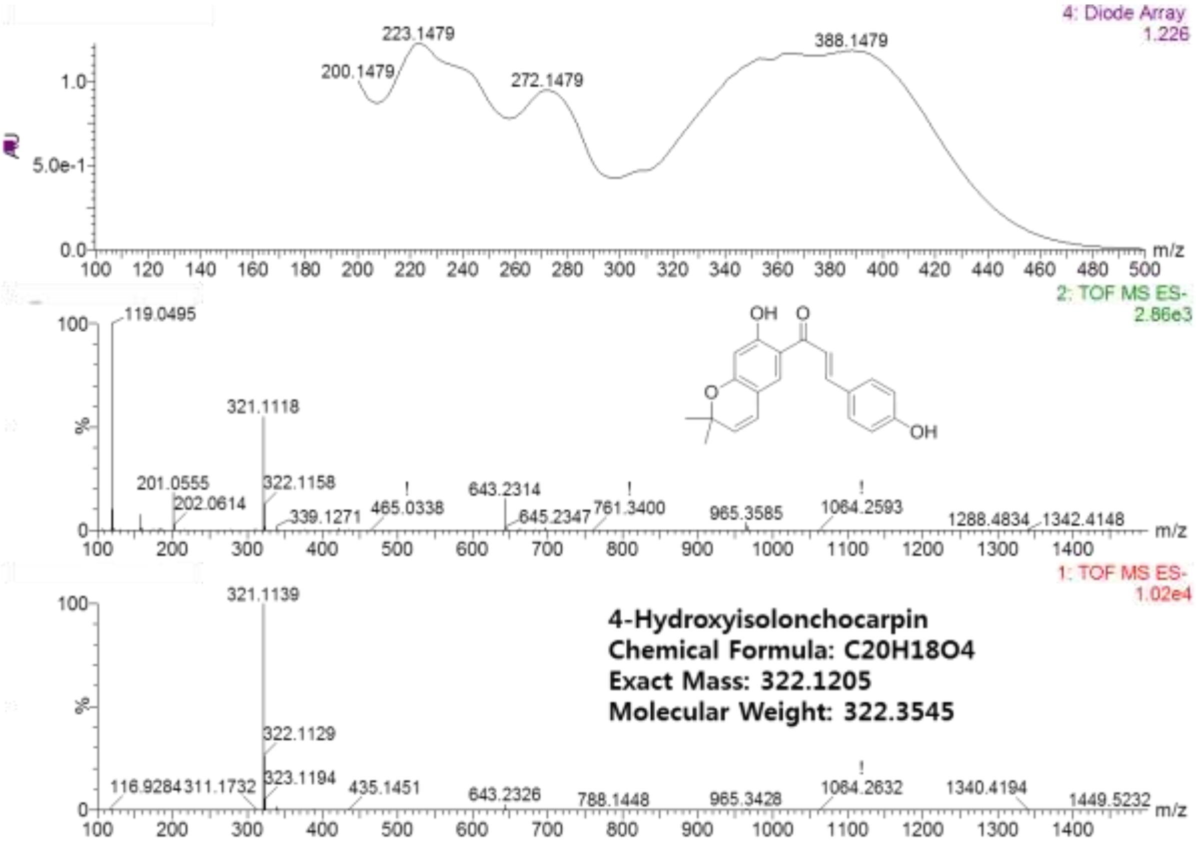

Single Mass Analysis
Tolerance = 10.0 PPM / DBE: min = -1.5, max = 50.0
Element prediction: Off
Number of isotope peaks used for i-FIT = 3
Monoisotopic Mass, Even Electron Ions
60 formula(e) evaluated with 1 results within limits (up to 50 best isotopic matches for each mass)
Elements Used:

Mass	Calc. Mass	mDa	PPM	DBE	Formula	i-FIT	i-FIT Norm	Fit Conf %	C	H	O
321.1139	321.1127	1.2	3.7	12.5	C20 H17 O4	101.6	n/a	n/a	20	17	4

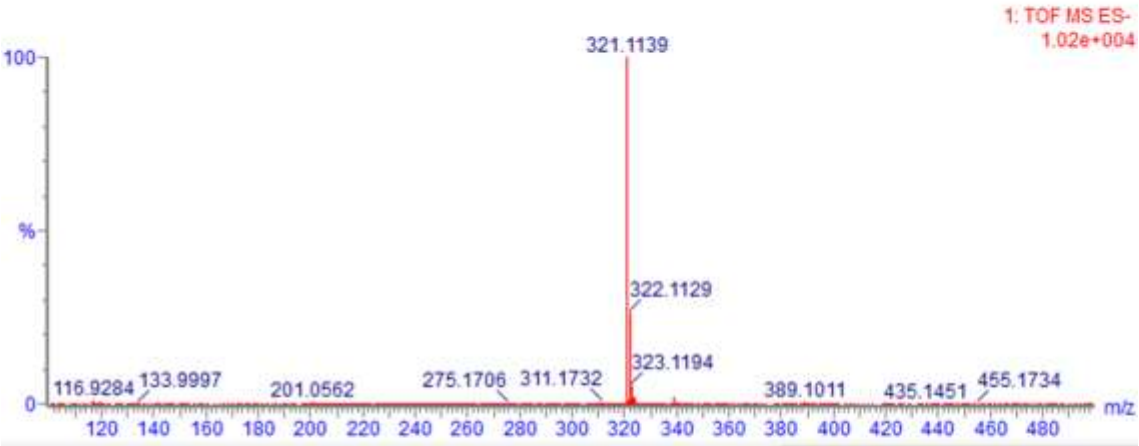

Figure S24. UPLC-QTOF-MS and HREIMS data of compound **3**.

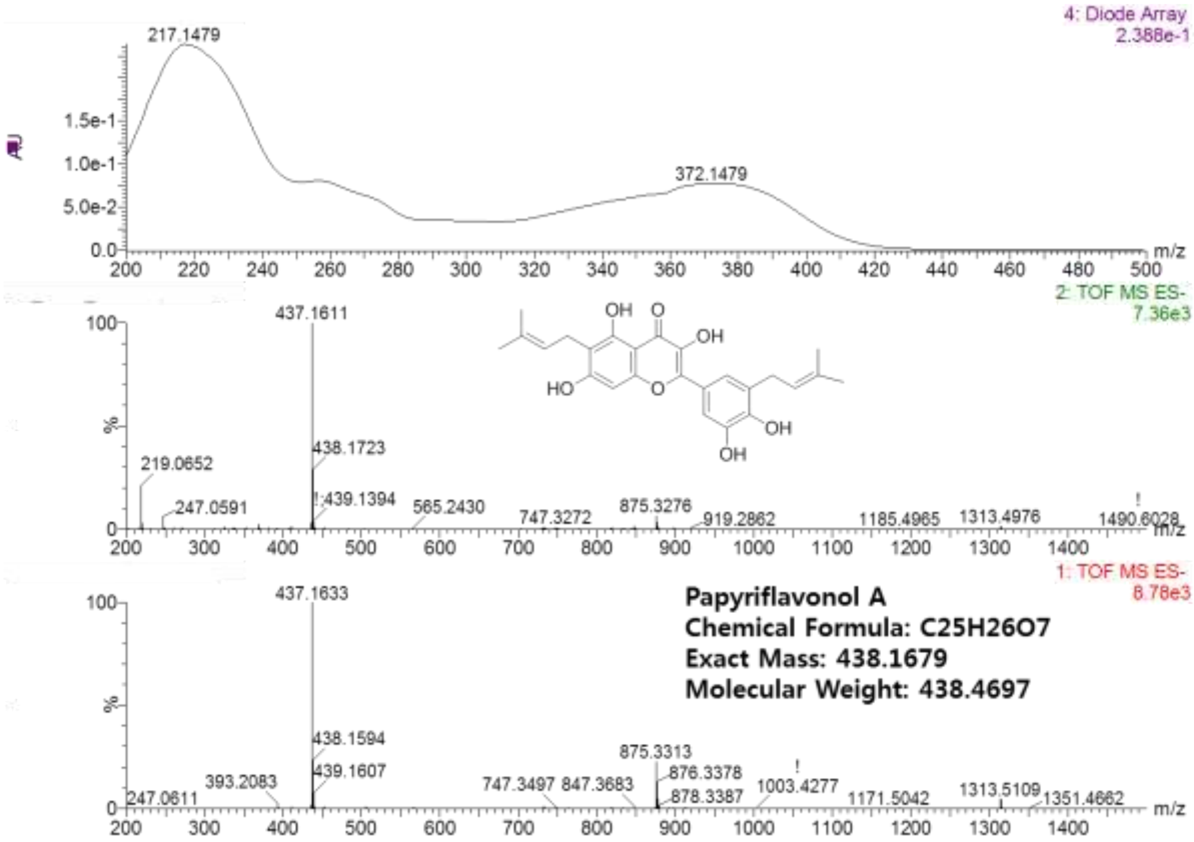

Single Mass Analysis
Tolerance = 10.0 PPM / DBE: min = -1.5, max = 50.0
Element prediction: Off
Number of isotope peaks used for i-FIT = 3
Monoisotopic Mass, Even Electron Ions
100 formula(e) evaluated with 2 results within limits (up to 50 best isotopic matches for each mass)
Elements Used:

Mass	Calc. Mass	mDa	PPM	DBE	Formula	i-FIT	i-FIT Norm	Fit Conf %	C	H	O
437.1633	437.1600	3.3	7.5	13.5	C25 H25 O7	161.8	0.680	50.68	25	25	7
	437.1659	-2.6	-5.9	4.5	C18 H29 O12	161.8	0.707	49.32	18	29	12

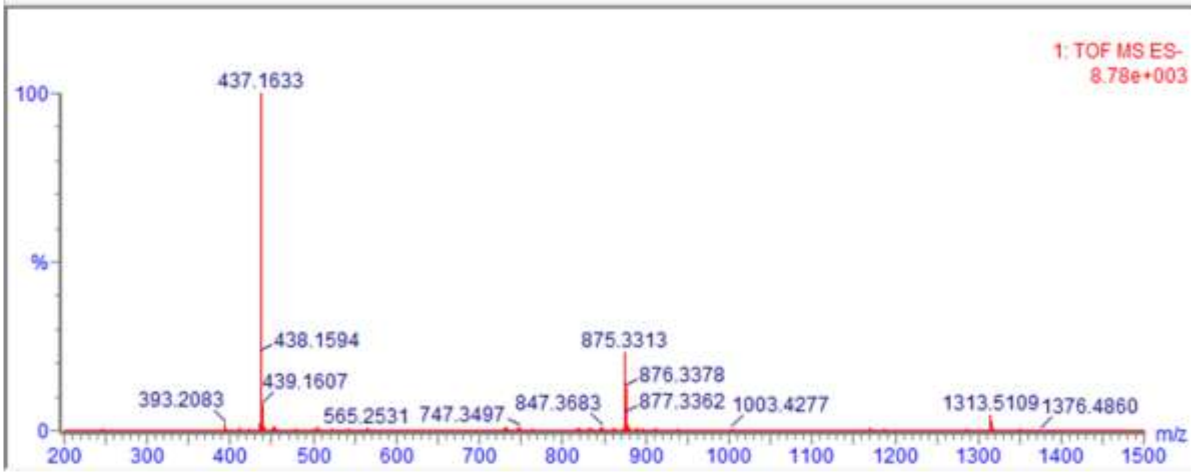

Figure S25. UPLC-QTOF-MS and HREIMS data of compound **4**.

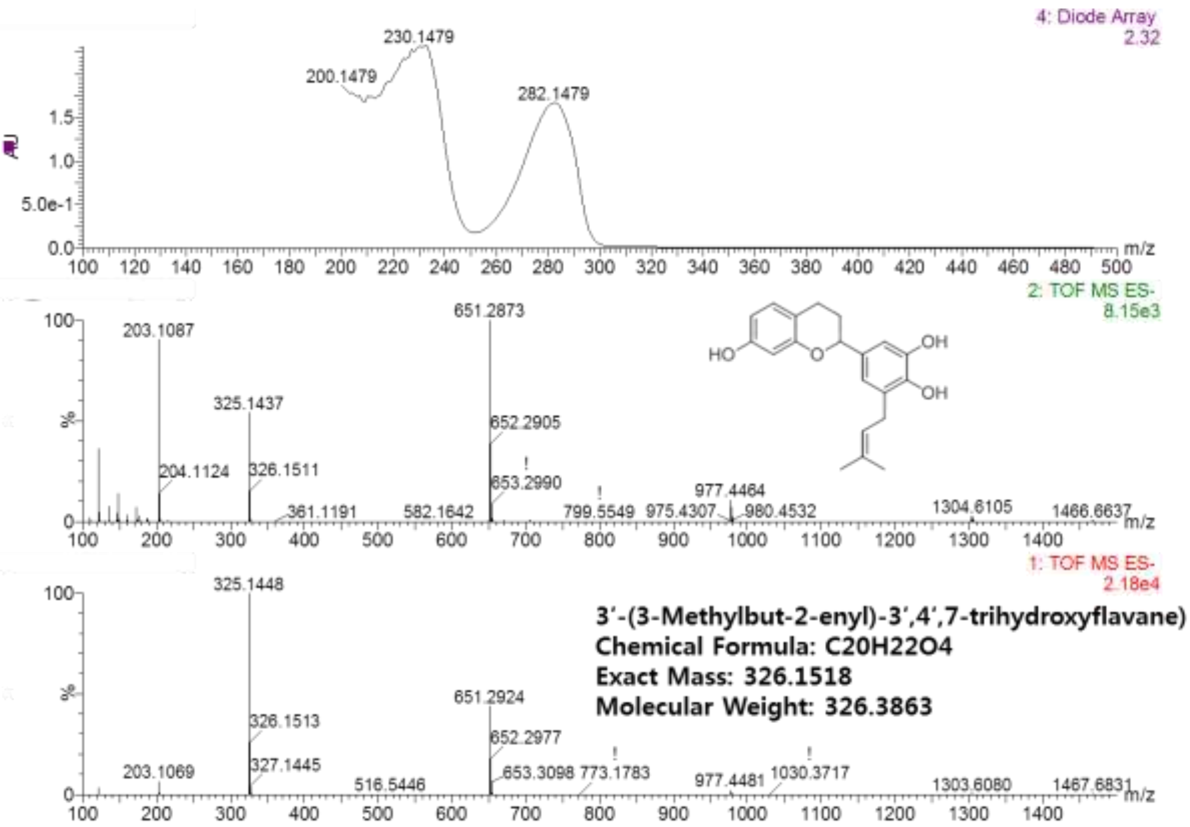

Single Mass Analysis

Tolerance = 10.0 PPM / DBE: min = -1.5, max = 50.0

Element prediction: Off

Number of isotope peaks used for i-FIT = 3

Monoisotopic Mass, Even Electron Ions

61 formula(e) evaluated with 1 results within limits (up to 50 best isotopic matches for each mass)

Elements Used:

Mass	Calc. Mass	mDa	PPM	DBE	Formula	i-FIT	i-FIT Norm	Fit Conf %	C	H	O
325.1448	325.1440	0.8	2.5	10.5	C20 H21 O4	218.1	n/a	n/a	20	21	4

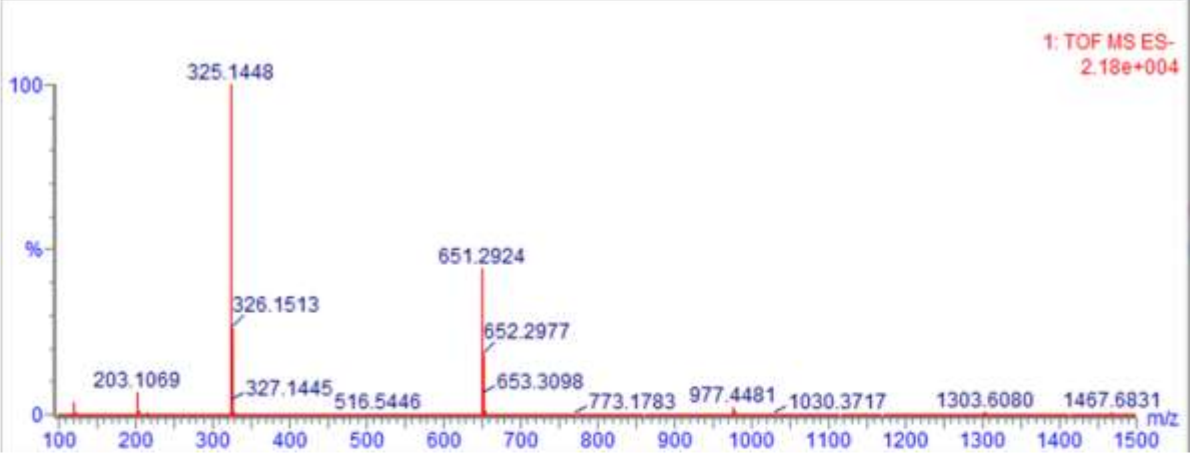

Figure S26. UPLC-QTOF-MS and HREIMS data of compound **5**.

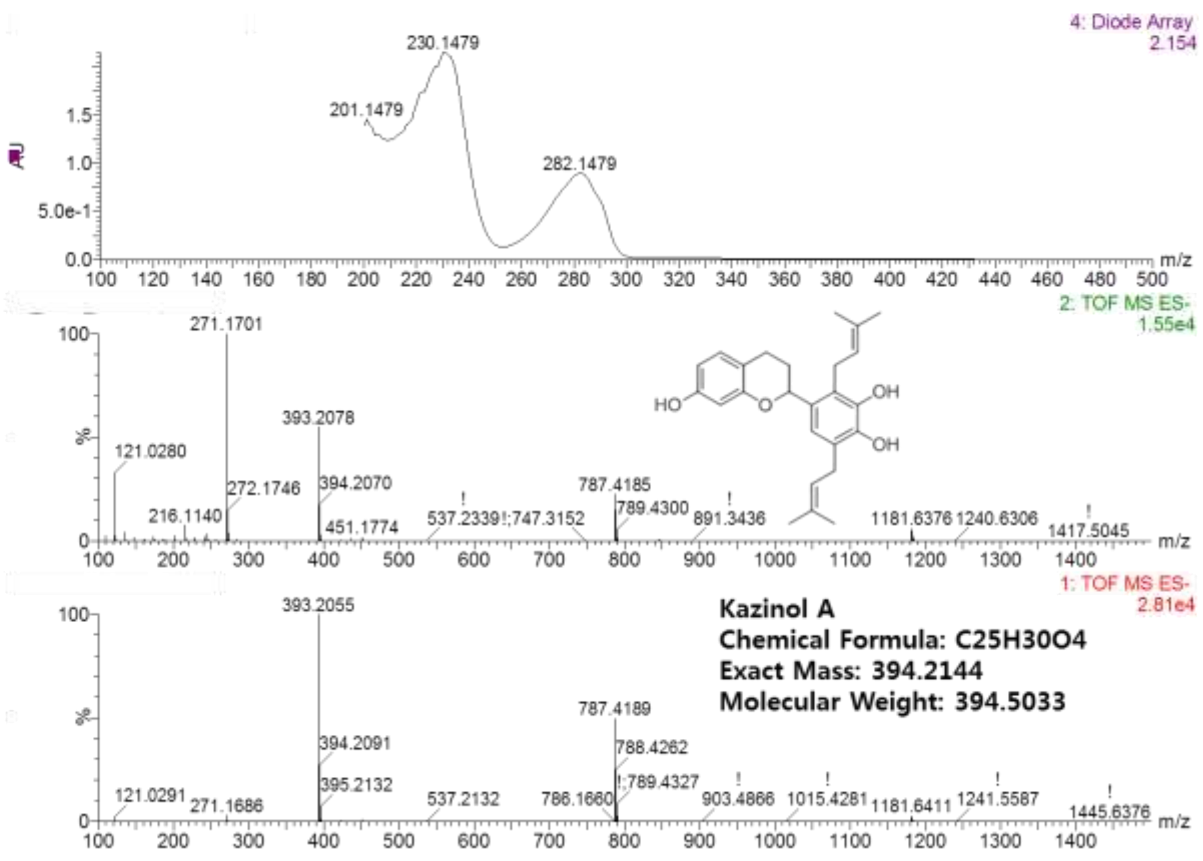

Single Mass Analysis
Tolerance = 10.0 PPM / DBE: min = -1.5, max = 50.0
Element prediction: Off
Number of isotope peaks used for i-FIT = 3
Monoisotopic Mass, Even Electron Ions
83 formula(e) evaluated with 1 results within limits (up to 50 best isotopic matches for each mass)
Elements Used:

Mass	Calc. Mass	mDa	PPM	DBE	Formula	i-FIT	i-FIT Norm	Fit Conf %	C	H	O
393.2055	393.2066	-1.1	-2.8	11.5	C25 H29 O4	230.0	n/a	n/a	25	29	4

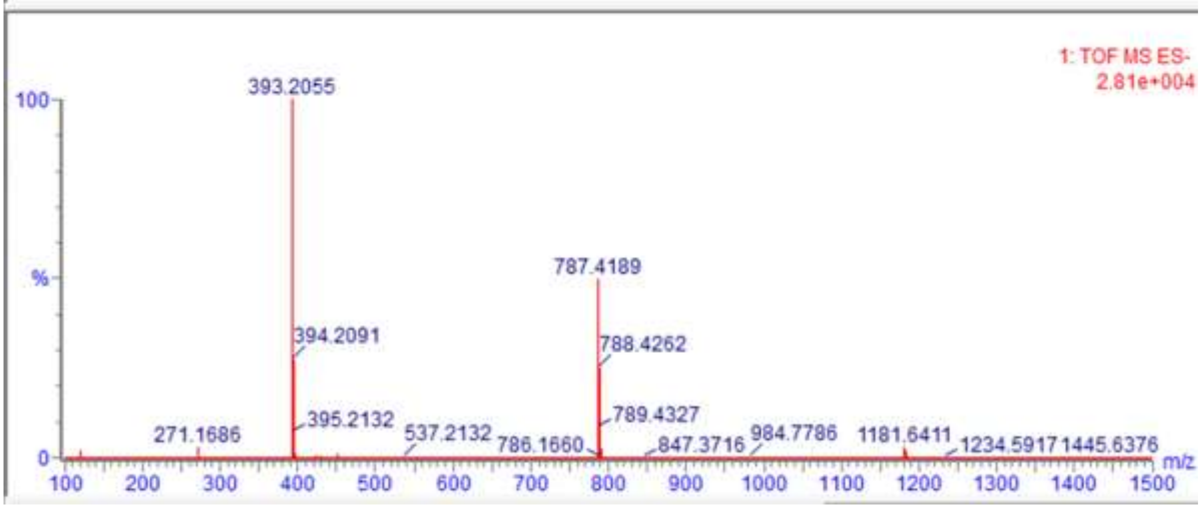

Figure S27. UPLC-QTOF-MS and HREIMS data of compound **6**.

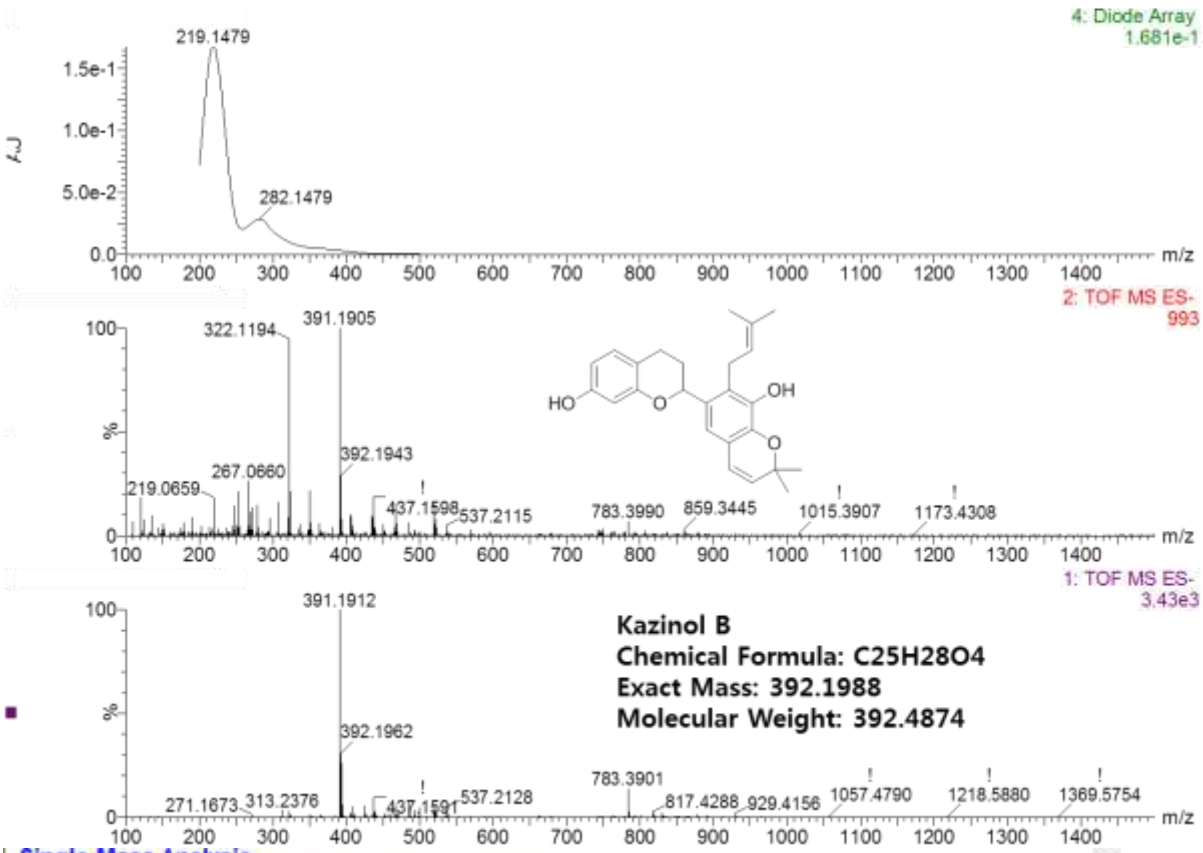

Single Mass Analysis

Tolerance = 10.0 PPM / DBE: min = -1.5, max = 50.0

Element prediction: Off

Number of isotope peaks used for i-FIT = 3

Monoisotopic Mass, Even Electron Ions

87 formula(e) evaluated with 1 results within limits (up to 50 best isotopic matches for each mass)

Elements Used:

Mass	Calc. Mass	mDa	PPM	DBE	Formula	i-FIT	i-FIT Norm	Fit Conf %	C	H	O
391.1912	391.1909	0.3	0.8	12.5	C25 H27 O4	162.2	n/a	n/a	25	27	4

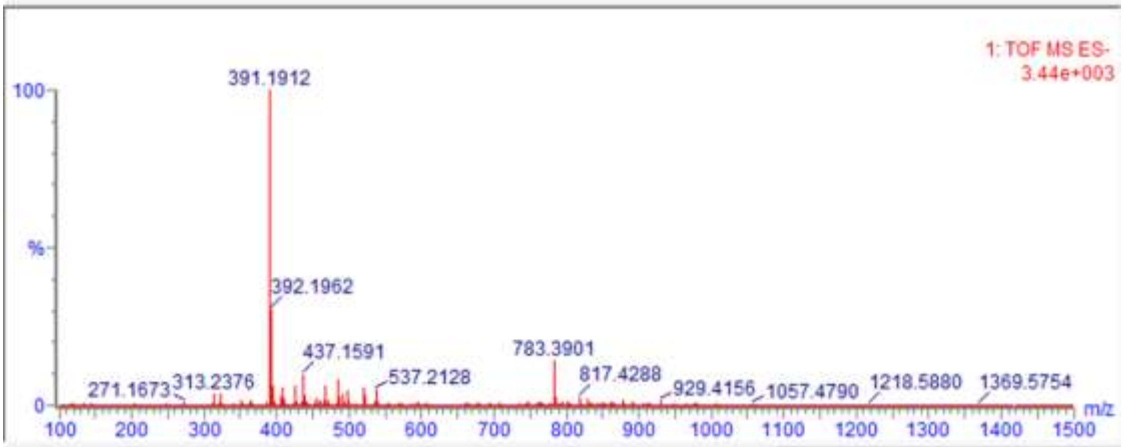

Figure S28. UPLC-QTOF-MS and HREIMS data of compound **7**.

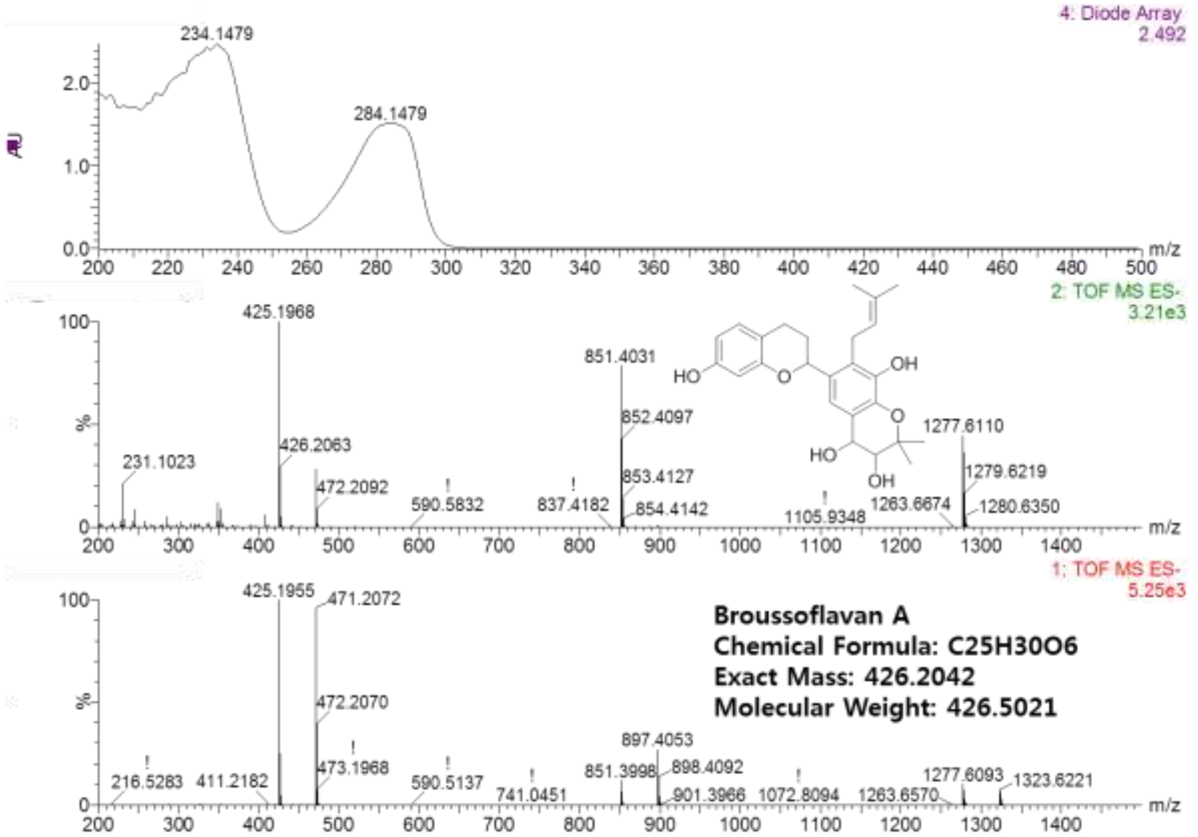

Single Mass Analysis
Tolerance = 10.0 PPM / DBE: min = -1.5, max = 50.0
Element prediction: Off
Number of isotope peaks used for i-FIT = 3
Monoisotopic Mass, Even Electron Ions
95 formula(e) evaluated with 1 results within limits (up to 50 best isotopic matches for each mass)
Elements Used:

Mass	Calc. Mass	mDa	PPM	DBE	Formula	i-FIT	i-FIT Norm	Fit Conf %	C	H	O
425.1955	425.1964	-0.9	-2.1	11.5	C25 H29 O6	70.9	n/a	n/a	25	29	6

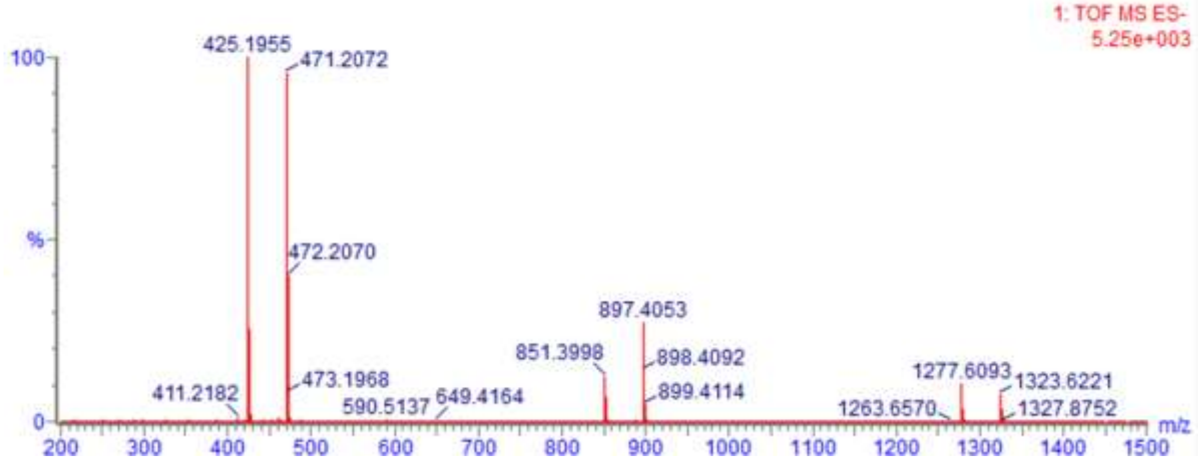

Figure S29. UPLC-QTOF-MS and HREIMS data of compound **8**.

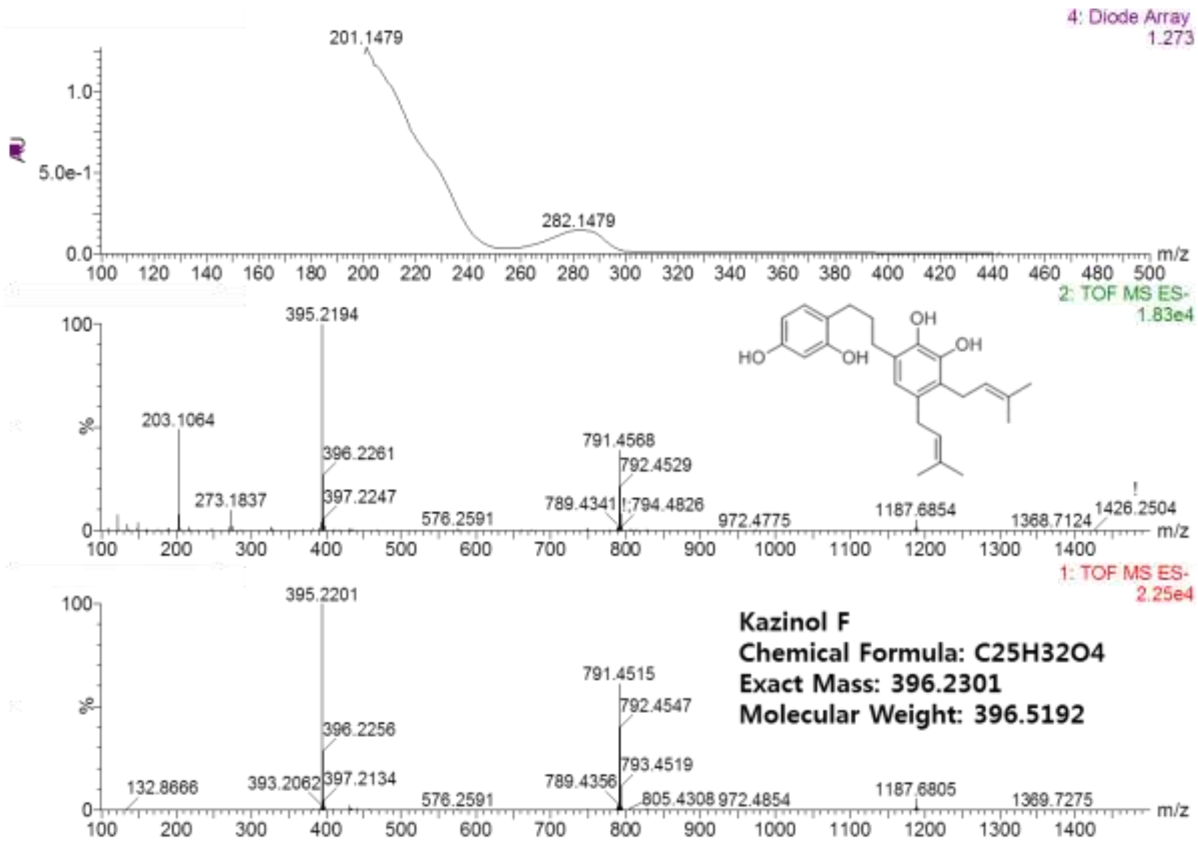

Single Mass Analysis
Tolerance = 10.0 PPM / DBE: min = -1.5, max = 50.0
Element prediction: Off
Number of isotope peaks used for i-FIT = 3
Monoisotopic Mass, Even Electron Ions
88 formula(e) evaluated with 1 results within limits (up to 50 best isotopic matches for each mass)
Elements Used:

Mass	Calc. Mass	mDa	PPM	DBE	Formula	i-FIT	i-FIT Norm	Fit Conf %	C	H	O
395.2201	395.2222	-2.1	-5.3	10.5	C25 H31 O4	246.4	n/a	n/a	25	31	4

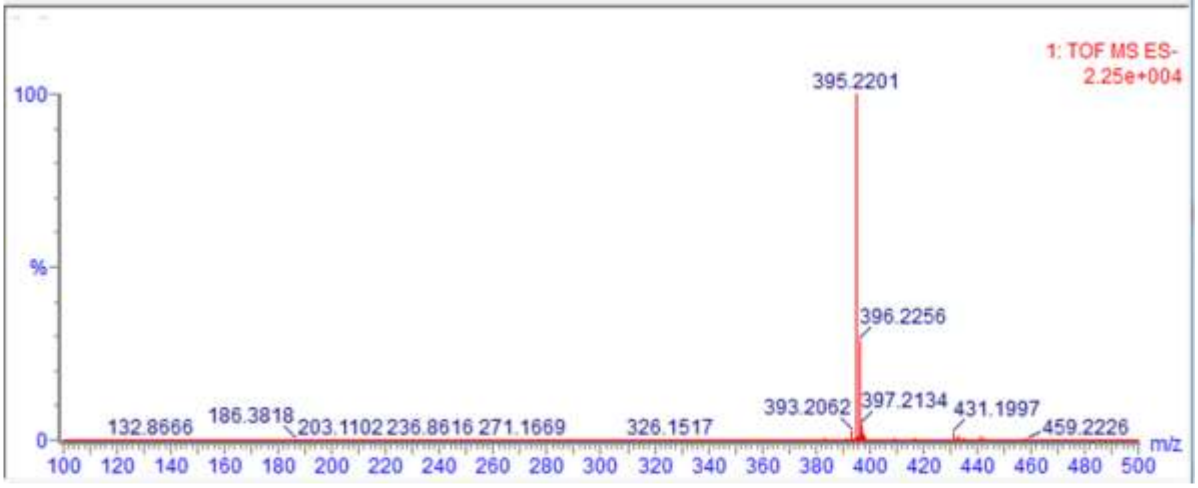

Figure S30. UPLC-QTOF-MS and HREIMS data of compound **9**.

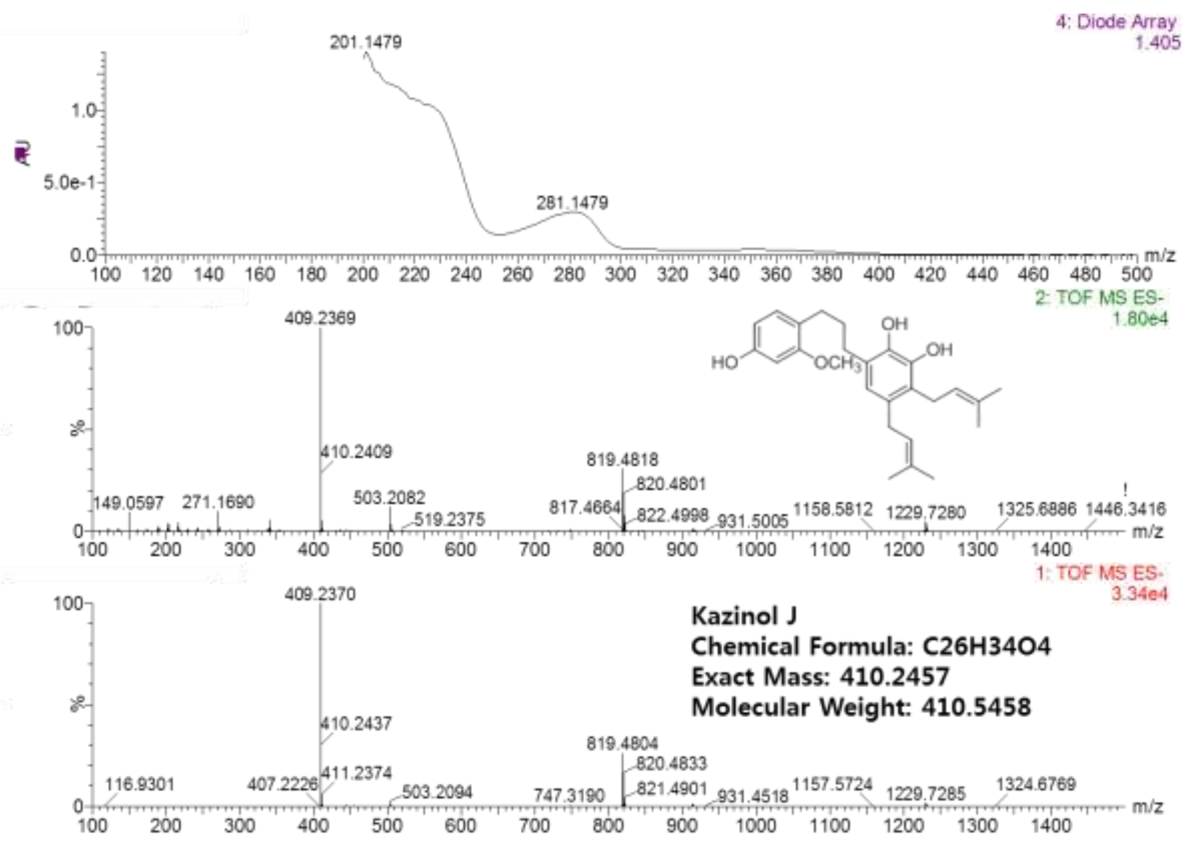

Single Mass Analysis
Tolerance = 10.0 PPM / DBE: min = -1.5, max = 50.0
Element prediction: Off
Number of isotope peaks used for i-FIT = 3
Monoisotopic Mass, Even Electron Ions
89 formula(e) evaluated with 1 results within limits (up to 50 best isotopic matches for each mass)
Elements Used:

Mass	Calc. Mass	mDa	PPM	DBE	Formula	i-FIT	i-FIT Norm	Fit Conf %	C	H	O
409.2370	409.2379	-0.9	-2.2	10.5	C26 H33 O4	221.3	n/a	n/a	26	33	4

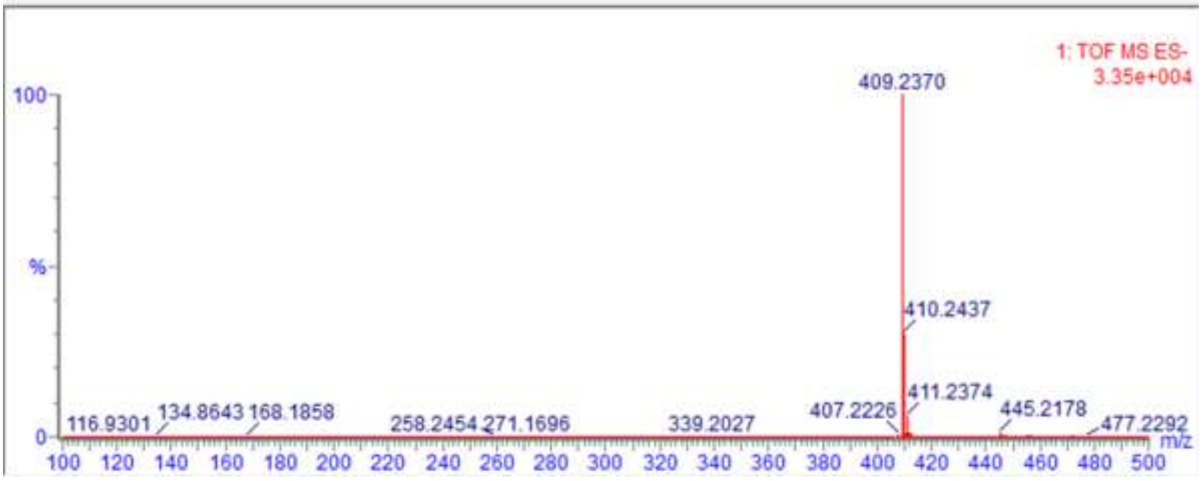

Figure S31. UPLC-QTOF-MS and HREIMS data of compound **10**.

```
1   agcggtttggtgaaaatgtcacatcccagtggagacgttgaggcttgtatggttcaggtt
    S  G  L  V  K  M  S  H  P  S  G  D  V  E  A  C  M  V  Q  V
61  acctgcggtagcatgactcttaatggtctttggcttgacaacacagtctggtgcccacga
    T  C  G  S  M  T  L  N  G  L  W  L  D  N  T  V  W  C  P  R
121 cacgtaatgtgcccggctgaccagttgtctgatcctaattatgatgccttgttgatttct
    H  V  M  C  P  A  D  Q  L  S  D  P  N  Y  D  A  L  L  I  S
181 atgactaatcatagtttcagtgtgcaaaaacacattggcgctccagcaaacttgcgtgtt
    M  T  N  H  S  F  S  V  Q  K  H  I  G  A  P  A  N  L  R  V
241 gttggtcatgccatgcaaggcactcttttgaagttgactgtcgatgttgctaaccctagc
    V  G  H  A  M  Q  G  T  L  L  K  L  T  V  D  V  A  N  P  S
301 actccagcctacacttttacaacagtgaaacctggcgcagcatttagtgtgttagcatgc
    T  P  A  Y  T  F  T  T  V  K  P  G  A  A  F  S  V  L  A  C
361 tataatggtcgtccgactggtacattcactgttgtaatgcgccctaactacacaattaag
    Y  N  G  R  P  T  G  T  F  T  V  V  M  R  P  N  Y  T  I  K
421 ggttcctttctgtgtggttcttgtggtagtgttggttacaccaaggagggtagtgtgatc
    G  S  F  L  C  G  S  C  G  S  V  G  Y  T  K  E  G  S  V  I
481 aattttgttacatgcatcaaatggaacttgctaatggtacacataccggttcagcattt
    N  F  C  Y  M  H  Q  M  E  L  A  N  G  T  H  T  G  S  A  F
541 gatggtactatgtatggtgcctttatggataaacaagtgcaccaagttcagttaacagac
    D  G  T  M  Y  G  A  F  M  D  K  Q  V  H  Q  V  Q  L  T  D
601 aaatactgcagtgttaatgtagtagcttggctttacgcagcaatacttaatggttgcgct
    K  Y  C  S  V  N  V  V  A  W  L  Y  A  A  I  L  N  G  C  A
661 tggtttgtaaaacctaatcgcactagtgttgtttcttttaatgaatgggctcttgccaac
    W  F  V  K  P  N  R  T  S  V  V  S  F  N  E  W  A  L  A  N
721 caattcactgaatttgttggcactcaatccgttgacatgttagctgtcaaaacaggcgtt
    Q  F  T  E  F  V  G  T  Q  S  V  D  M  L  A  V  K  T  G  V
781 gctattgaacagctgctttatgcgatccaacaactttatactgggttccagggaaagcaa
    A  I  E  Q  L  L  Y  A  I  Q  Q  L  Y  T  G  F  Q  G  K  Q
841 atccttggcagtaccatgttggaagatgaattcacacctgaggatgttaatatgcagatt
    I  L  G  S  T  M  L  E  D  E  F  T  P  E  D  V  N  M  Q  I
901 atgggtgtggttatgcag
    M  G  V  V  M  Q
```

Figure S32. Nucleotide sequence of the synthetic MERS-CoV 3CL^{pro} gene. The synthetic gene is based on the amino acid sequence of MERS-CoV complete genome (GenBank accession number KF192507.1: polyprotein residues 3247-3553)

```
1   cagttaacaatcgaagtcttagtgactgtcgatggtgtaaattttagaacagtcgttcta
    Q  L  T  I  E  V  L  V  T  V  D  G  V  N  F  R  T  V  V  L
61  aataataagaacacttatagatcacagcttggatgcgttttctttaatggtgctgatatt
    N  N  K  N  T  Y  R  S  Q  L  G  C  V  F  F  N  G  A  D  I
121 tctgacaccattcctgatgagaaacagaatggtcacagtttatatctagcagacaatttg
    S  D  T  I  P  D  E  K  Q  N  G  H  S  L  Y  L  A  D  N  L
181 actgctgatgaaacaaaggcgcttaaagagttatatggccccgttgatcctactttctta
    T  A  D  E  T  K  A  L  K  E  L  Y  G  P  V  D  P  T  F  L
241 cacagattctattcacttaaggctgcagtccataagtggaagatggttgtgtgtgataag
    H  R  F  Y  S  L  K  A  A  V  H  K  W  K  M  V  V  C  D  K
301 gtacgttctctcaaattgagtgataataattgttatcttaatgcagttattatgacactt
    V  R  S  L  K  L  S  D  N  N  C  Y  L  N  A  V  I  M  T  L
361 gatttattgaaggacattaaatttgttatacctgctctacagcatgcatttatgaaacat
    D  L  L  K  D  I  K  F  V  I  P  A  L  Q  H  A  F  M  K  H
421 aagggcggtgattcaactgacttcatagccctcattatggcttatggcaattgcacattt
    K  G  G  D  S  T  D  F  I  A  L  I  M  A  Y  G  N  C  T  F
481 ggtgctccagatgatgcctctcggttacttcataccgtgcttgcaaaggctgagttatgc
    G  A  P  D  D  A  S  R  L  L  H  T  V  L  A  K  A  E  L  C
541 tgttctgcacgcatggtttggagagagtggtgcaatgtctgtggcataaaagatgttgtt
    C  S  A  R  M  V  W  R  E  W  C  N  V  C  G  I  K  D  V  V
601 ctacaaggcttaaaagcttgttgttacgtgggtgtgcaaactgttgaagatctgcgtgct
    L  Q  G  L  K  A  C  C  Y  V  G  V  Q  T  V  E  D  L  R  A
661 cgcatgacatatgtatgccagtgtggtggtgaacgtcatcggcaaatagtcgaacacacc
    R  M  T  Y  V  C  Q  C  G  G  E  R  H  R  Q  I  V  E  H  T
721 acccccctggttgctgctctcaggcacaccaaatgaaaaattggtgacaacctccacggcg
    T  P  W  L  L  L  S  G  T  P  N  E  K  L  V  T  T  S  T  A
781 cctgattttgtagcgtttaatgtctttcagggcattgaaacggctgttggccattatgtt
    P  D  F  V  A  F  N  V  F  Q  G  I  E  T  A  V  G  H  Y  V
841 catgctcgcctgaagggtggtcttattttaaagtttgactctggcaccgttagcaagact
    H  A  R  L  K  G  G  L  I  L  K  F  D  S  G  T  V  S  K  T
901 tcagactggaagtgcaaggtgacagatgtacttttccccggccaaaaatacagtagcgat
    S  D  W  K  C  K  V  T  D  V  L  F  P  G  Q  K  Y  S  S  D
```

Figure S33. Nucleotide sequence of the synthetic MERS-CoV PL^{pro} gene. The synthetic gene is based on the amino acid sequence of MERS-CoV complete genome (GenBank accession number KF192507.1: polyprotein residues 1436-1803)

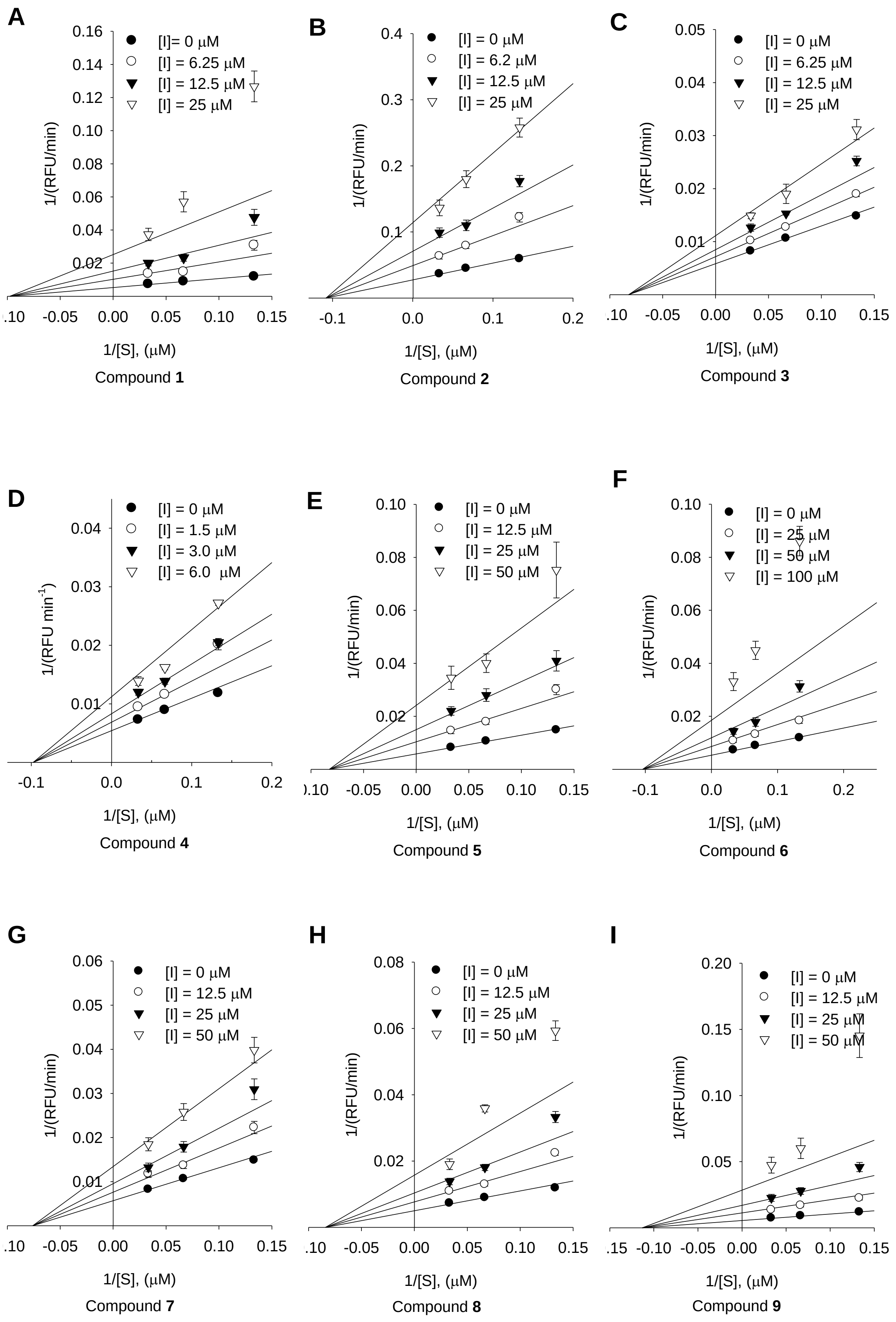
A
[I]= 0 μM
[I] = 6.25 μM
[I] = 12.5 μM
[I] = 25 μM
1/(RFU/min)
1/[S], (μM)
Compound 1
B
[I] = 0 μM
[I] = 6.2 μM
[I] = 12.5 μM
[I] = 25 μM
1/(RFU/min)
1/[S], (μM)
Compound 2
C
[I] = 0 μM
[I] = 6.25 μM
[I] = 12.5 μM
[I] = 25 μM
1/(RFU/min)
1/[S], (μM)
Compound 3
D
[I] = 0 μM
[I] = 1.5 μM
[I] = 3.0 μM
[I] = 6.0 μM
1/(RFU min-1)
1/[S], (μM)
Compound 4
E
[I] = 0 μM
[I] = 12.5 μM
[I] = 25 μM
[I] = 50 μM
1/(RFU/min)
1/[S], (μM)
Compound 5
F
[I] = 0 μM
[I] = 25 μM
[I] = 50 μM
[I] = 100 μM
1/(RFU/min)
1/[S], (μM)
Compound 6
G
[I] = 0 μM
[I] = 12.5 μM
[I] = 25 μM
[I] = 50 μM
1/(RFU/min)
1/[S], (μM)
Compound 7
H
[I] = 0 μM
[I] = 12.5 μM
[I] = 25 μM
[I] = 50 μM
1/(RFU/min)
1/[S], (μM)
Compound 8
I
[I] = 0 μM
[I] = 12.5 μM
[I] = 25 μM
[I] = 50 μM
1/(RFU/min)
1/[S], (μM)
Compound 9

J

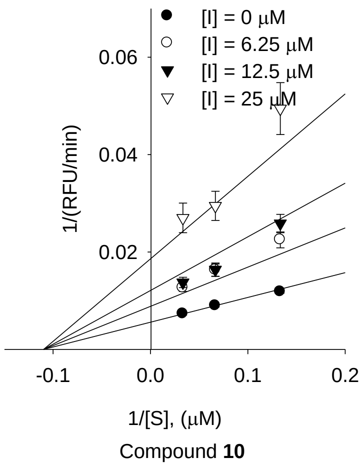

Figure S34. Lineweaver-Burk plots for inhibition of compounds(**1-10**) on SARS-CoV PL^{pro}.
